# Supplementary material for: Modern contraceptive use among sexually active women aged 15–19 years in North-Western Tanzania: results from the Adolescent 360 (A360) baseline survey
Source: BMJ Open. 2019 Aug 28;9(8):e030485. doi: 10.1136/bmjopen-2019-030485 (PMC6720144; doi:10.1136/bmjopen-2019-030485)
Supplement: Author's manuscript [file bmjopen-2019-030485.draft_revisions.pdf]

BMJ Open

BMJ Open is committed to open peer review. As part of this commitment we make the peer review history of every article we publish publicly available.

When an article is published we post the peer reviewers' comments and the authors' responses online. We also post the versions of the paper that were used during peer review. These are the versions that the peer review comments apply to.

The versions of the paper that follow are the versions that were submitted during the peer review process. They are not the versions of record or the final published versions. They should not be cited or distributed as the published version of this manuscript.

BMJ Open is an open access journal and the full, final, typeset and author-corrected version of record of the manuscript is available on our site with no access controls, subscription charges or pay-per-view fees (http://bmjopen.bmj.com).

If you have any questions on BMJ Open's open peer review process please email info.bmjopen@bmj.com

BMJ Open

Factors associated with modern contraceptive use among sexually active women aged 15 to 19 years in north-western Tanzania: results from the Adolescent 360 (A360) baseline survey

Journal:	*BMJ Open*
Manuscript ID	bmjopen-2019-030485
Article Type:	Research
Date Submitted by the Author:	20-Mar-2019

Complete List of Authors:	Nsanya, Mussa; Mwanza Intervention Trials Unit,<br>Atchison, Christina; London School of Hygiene and Tropical Medicine Faculty of Epidemiology and Population Health, MRC Tropical Epidemiology Group<br>Bottomley, Christian ; London School of Hygiene and Tropical Medicine Faculty of Epidemiology and Population Health, MRC Tropical Epidemiology Group<br>Doyle, Aoife; London School of Hygiene and Tropical Medicine Faculty of Epidemiology and Population Health, MRC Tropical Epidemiology Group<br>Kapiga, Saidi ; London School of Hygiene and Tropical Medicine, Infectious Disease Epidemiology; London School of Hygiene and Tropical Medicine Faculty of Epidemiology and Population Health, MRC Tropical Epidemiology Group

Keywords:	Adolescents, Contraception, Family Planning, Reproductive health, Africa

SCHOLARONE™
Manuscripts

TITLE

Factors associated with modern contraceptive use among sexually active women aged 15 to 19 years in north-western Tanzania: results from the Adolescent 360 (A360) baseline survey

Key words: Adolescents, Contraception, Family Planning, Reproductive health, Africa

Authors:

Mussa Kelvin Nsanya[1*] , Christina J. Atchison[2], Christian Bottomley[2], Aoife M. Doyle[2], and Saidi H. Kapiga[1,3]

Institutional Affiliations:

1.Mwanza Intervention Trials Unit/National Institute for Medical Research, P.O. Box 11936, Mwanza, Tanzania

2. MRC Tropical Epidemiology Group, London School of Hygiene and Tropical Medicine, Keppel Street, London WC1E 7H, UK

3. Department of Infectious Disease Epidemiology, London School of Hygiene and Tropical Medicine, Keppel Street, London WC1E 7H, UK

*Corresponding author: mussa.nsanya@mitu.or.tz / kelvin.nsanya@gmail.com

For peer review only

ABSTRACT

Objectives: To describe sexuality, fertility and family planning characteristics of adolescent women aged 15 to 19 years and to determine factors associated with modern contraceptive use among sexually active women in this population.

Design: Cross-sectional analysis of Adolescent 360 (A360) evaluation baseline survey.

Setting: 15 randomly chosen urban and semi-urban wards of Ilemela district, in Mwanza region at north-western Tanzania.

Participants: Adolescent women aged 15 – 19 years who were living in the study site from August 2017 to February 2018 and who provided informed consent. They were classified as married if they had a husband or were living as married. Unmarried women were classified as sexually active if they reported having sexual intercourse in past 12 months.

Outcome measure: Prevalence of modern contraceptive (mCPR) among adolescent women aged 15 – 19 years.

Results: Data was available for 3,511 women aged 15-19 years, of which 201(5.7%) were married and 744 (22.5%) were unmarried-sexually active. We found strong evidence of differences between married and unmarried-sexually active women across socio-demographic, fertility and contraception use characteristics. Determinants of modern contraception use among unmarried-sexually active women were increasing age, increasing level of education, being in education, hearing of modern contraception from interpersonal sources or in the media in the past 12 months, perceiving partner and/or friends support for contraceptive use, as well as higher knowledge and self efficacy for contraception.

Conclusions: Sexual and reproductive health programmes aiming to increase uptake of modern contraceptives should consider the importance of girl's education and social support for contraceptive use among unmarried-sexually active adolescent women

For peer review only - http://bmjopen.bmj.com/site/about/guidelines.xhtml

For peer review only

Strengths and limitations of this study

- We used the probability sampling approach to obtain 3,511 adolescent women from 34 streets in the 15 urban and semi-urban wards of Ilemela district, Mwanza. This approach ensured that these findings are generalizable to the wider population of adolescent women aged 15 to 19 years living in similar urban and peri-urban wards of other regions in Tanzania where A360 intervention is being rolled out.
- The main limitations are: its design being cross-sectional makes temporal causal relationships hard to establish and reverse causality is likely, for example, use of contraceptives may lead to higher knowledge about contraceptives.
- Also, because of the small sample size of married women, the study had limited power to identify determinants for contraceptive use in this group and hence the decision to drop them from the analysis.

INTRODUCTION

Globally, approximately 16 million women aged 15 to 19 years give birth each year and 95% of these births take place in low and middle income countries.[1 2] The global community, through the Family Planning 2020 (FP2020) initiative, is committed to increase new contraceptive users to 120 million in 69 developing countries (including Tanzania) by 2020. [3] This initiative would also support the objectives of the United Nation's Sustainable Development Goal (SDG) 3 on health and wellbeing for all and SDG 5 on gender equality which also embodies sexual and reproductive health at the heart of global efforts to sustainable development particularly in low and middle income countries.[4]

For peer review only - http://bmjopen.bmj.com/site/about/guidelines.xhtml

Modern contraceptive use remains low in sub-Saharan Africa despite increasing awareness and knowledge about contraception.[5 6] For instance, 69% and 59% of the women aged 15 to 19 years in United Kingdom and United States of America respectively report using a modern contraceptive at the last time they had sexual intercourse compared to 12% in Mali and 21% in Tanzania.[7] The low uptake of modern contraceptives particularly among women aged 15 to 19 years contributes significantly to high rates of adolescent pregnancies and poor health outcomes including maternal morbidity and mortality, and neonatal and under-five child mortality.[1 5 8] In addition, there are other severe social and economic consequences to adolescent women, their families and whole society including not reaching their potential for educational achievement, and not getting a paid job which usually leads into a vicious cycle of poverty. [1 9]

Most studies to date have focused on the factors that prevent women of reproductive age (15 – 49 years) from using modern contraceptives.[5] In such studies adolescent women are usually underrepresented despite facing disproportionate medical, social and economic impact of unintended pregnancies.[1] In order for the goals of FP2020 and SDG 3 and 5 to be achieved, more information is required from studies which have examined factors associated with contraceptive use among adolescent women in developing countries, including Tanzania.

Adolescents 360 (A360) is an initiative being rolled out across Ethiopia, Nigeria and Tanzania, aiming to increase uptake of voluntary modern contraception among sexually active women aged 15 to 19 years.[10] Using baseline survey data collected as part of A360 programme evaluation, we describe sexuality, fertility and family planning characteristics of adolescent women aged 15 to 19 years; and determine factors associated with modern contraceptive use among sexually active women in this population in Mwanza, Tanzania.

METHODS

For peer review only - http://bmjopen.bmj.com/site/about/guidelines.xhtml

For peer review only

Study design and settings

Between August 2017 and February 2018, we conducted a cross-sectional baseline survey among women aged 15 to 19 years in Mwanza city, Tanzania. The survey was part of a comprehensive outcome evaluation to assess the impact of the A360 programme on a number of sexual and reproductive health outcomes, primarily uptake of voluntary modern contraception among sexually active women aged 15 to 19 years.[10]

In Tanzania, A360 is being implemented in 16 administrative regions. This survey was conducted in fifteen urban and semi-urban wards of Ilemela district, Mwanza region.[10] Ilemela district covers the northern part of Mwanza city and is comprised of 19 wards, of which four are rural wards. Each ward is administratively divided into a number of neighbourhoods, called 'streets'.

Study population

Women were included in the study if they were 15 to 19 years old; living in the study sites at the time of the survey; and voluntarily provided informed consent. Women were classified as married if they reported that they had a husband or were living as married with a cohabiting male partner.

Informed consent

Written informed consent was obtained from all participants. A parental consent waiver was granted for unmarried women aged 15 to 17 years, given the sensitive nature of the topics discussed. Married women under 18 years of age were considered emancipated and did not require parental consent in addition to their own voluntary consent.

Ethics approval

For peer review only - http://bmjopen.bmj.com/site/about/guidelines.xhtml

The study was approved by the Tanzania National Health Research Ethics review sub-committee of the National Institute for Medical Research (Ref: NIMR/HQ/R8a/Vol.IX/2549), and the research ethics committee of the London School of Hygiene & Tropical Medicine (LSHTM, Ref: 14145).

Sampling strategy and sample size

A cluster sampling design was used. The primary sampling unit (PSU) for the survey was a 'street', the smallest administrative unit similar to a neighbourhood or a localised and delineated group of people. All 15 urban or semi-urban wards of Ilemela district were included in the survey. Each ward has an estimated eight to ten streets. A simple random sample of 34 'streets' was selected across the 15 urban and semi-urban wards of Ilemela district. As per study protocol, in the first eight 'streets', we randomly selected 50 GPS coordinates using ArcGIS software version 9.3 (Esri, Redlands, USA). All households whose front door was located within a radius of 20 meters around the GPS coordinates were visited and all eligible consenting women aged 15 to 19 years residing in these households were invited to be interviewed. Fewer eligible women than predicted were surveyed using this sampling strategy, thus in the remaining 26 'streets' we visited all households and administered the questionnaire to all eligible and consenting women aged 15 to 19 years. Our target sample size for the baseline survey was 3,314 women aged 15 to 19 years.[10]

If potentially eligible participants were not available at the first visit, two further revisits were made to attempt to hold interviews.

Participants and Public Involvement

We sought permission from local government authorities in the wards where the study took place as well as from individual participants prior to enrollment. Additionally, we have communicated the A360 baseline survey report to local government officials in Ilemela district, Mwanza

For peer review only

Tool for baseline survey

The questionnaire was adapted from various research instruments that have been used in the target countries including the Tanzania Demographic and Health Survey (DHS)[11] and FP2020 survey instruments[3]. Questionnaires were administered face-to-face by female interviewers aged between 18 and 26 years using pre-programmed tablet computers.[10]

The questionnaire had three components: (1) socio-demographic characteristics, (2) fertility characteristics and preferences, and (3) contraceptive knowledge, attitudes and practices. Only respondents who reported sexual intercourse in the last 12 months were considered sexually active hence asked questions about contraceptive use.[10]

Study outcome

The prevalence of modern contraceptives (mCPR) among married women aged 15 to 19 years was defined as per the DHS definition[12]:

$$\frac{\text{Number of married 15–19-year-old women reporting use of modern contraceptives at the time of the survey}}{\text{Number of married 15–19-year-old women}}$$

mCPR among unmarried-sexually active women aged 15 to 19 years was defined as follows:

$$\frac{\text{Number of unmarried-sexually active* women aged 15–19-year-old reporting use of modern contraceptives at the time of the survey}}{\text{Number of unmarried-sexually active* unmarried women aged 15–19-year-old}}$$

For peer review only - http://bmjopen.bmj.com/site/about/guidelines.xhtml

For peer review only

*self-reported that they were sexually active in the 12 months prior to the survey

Modern contraception was defined to include the following: male and female sterilisation, contraceptive implants, intrauterine contraceptive devices (IUCD), injectables, oral contraceptive pill, emergency contraceptive pill, male condom, female condom, Standard Days Method (SDM), Lactational Amenorrhoea Method (LAM), diaphragm, spermicides, foams and jelly.

Statistical analysis

Data analysis was conducted in Stata 15. We used sampling weights and robust standard errors to account for the clustered sampling design.

Descriptive data analysis was done for both married and unmarried women. Logistic regression was performed for unmarried-sexually active women only due to small sample size for married women. We obtained odds ratios (OR) for the association of each explanatory variable with use of modern contraception. Wald tests adjusted for the clustered sampling design were used at each step of the analysis. The associations between mCPR and age and between mCPR and religion were not adjusted for other explanatory variables. Age and religion were considered a priori potential confounders for the associations between mCPR and highest education level achieved, currently being in education and socioeconomic position. The remaining explanatory variables with p-value < 0.2 in the univariate analysis, were investigated one-by-one in multivariate regression models adjusted for age, religion, highest education level achieved, currently being in education and socioeconomic position. Variables with p value < 0.05 in the adjusted analysis were considered to be associated with mCPR. This strategy allowed us to assess the effect of variables adjusted for distal a priori potential confounders.

For peer review only - http://bmjopen.bmj.com/site/about/guidelines.xhtml

Socioeconomic position was created from a series of questions about household items, dwelling materials and access to a bank account. The variable was generated using the "Tanzania Equity Tool" which uses different weights attached to each answer to create a composite score which was then split into quintiles according to the national thresholds.[13]

Knowledge about contraception was assessed through the respondents affirmative report to the following five questions: (1) preventing unintended pregnancies is a benefit of contraception, (2) preventing abortions is a benefit of contraception, (3) some contraceptive methods reduce sexually transmitted infections/HIV, (4) modern contraception can help with child spacing, and (5) using modern contraception can allow a woman to complete her education, take up better economic opportunities and fulfil her potential.

Holding misconceptions was assessed by asking respondents whether they agreed with the following four statements: (1) use of a long-acting reversible contraceptive can make adolescent women permanently infertile, (2) changes to normal menstrual bleeding patterns, which is caused by some contraceptives, are harmful to health, (3) modern contraceptives can make adolescent women permanently fat, and (4) adolescent women who use family planning/birth spacing may become promiscuous.

Self-efficacy for contraception was assessed through four questions relating to the woman's ability to access and use contraception: (1) felt able to start a conversation with her partner about contraception, (2) felt able to use a method of contraception even if her partner did not want her to, (3) felt able to obtain information on contraception services and products if she needed to, and (4) felt able to obtain a contraceptive method if she decided to use one.

For peer review only

Variables for contraception knowledge, holding misconceptions, and self-efficacy were created as scores from 0 - 5 for knowledge, and 0 - 4 for holding misconceptions and self-efficacy based on the overall score for each individual statement in each category. A score of 1 was given if the respondent agreed with the statement and 0 if she disagreed or answered "*don't know*". A maximum score of 5 for knowledge and 4 for self-efficacy would indicate that the respondent correctly agreed with all five knowledge statements and felt able to achieve all four self-efficacy behaviours. A maximum score of 4 for holding misconceptions would be interpreted as believing all four myth statements about contraception.

RESULTS

A total of 14,138 households were identified and 99.6% were interviewed to obtain information on household members. A total of 5,121 potentially eligible women aged 15–19 years were identified from 3,963 households (28.1% of all interviewed households); 68.6% (3,511) of potentially eligible women were interviewed, of whom 5.7% (201/3,511) were married. Overall, 22.5% (744/3,310) of unmarried women had been sexually active during the 12 months preceding the survey. The most common reason for a potentially eligible woman not being interviewed was that she was absent or unavailable after a maximum of three visits.

In Table 1, we present the characteristic of married and unmarried-sexually active women aged 15 – 19 years in the study population. The median age was 19 years for married women and 18 years for unmarried-sexually active women. The majority of respondents were Christian (married: 79.7%, unmarried: 84.5%) and were not currently pursuing educational training (married: 99.0%, unmarried: 79.6%). The highest level of education achieved by majority of married women was primary education (65.2%) while the majority of unmarried-sexually active women had achieved secondary level education (52.7%). Most respondents had moderate knowledge about contraception (married: 56.2%, unmarried: 62.4%) and a moderate self-efficacy for contraception (married: 53.0%,

For peer review only - http://bmjopen.bmj.com/site/about/guidelines.xhtml

unmarried: 56.7%). Overall, there was strong evidence of differences between married and unmarried-sexually active women across many of the measured characteristics (Table 1).

In Table 2, we present the prevalence of contraceptive use among women aged 15 – 19 years in Mwanza by their marital status. Overall, 19.4% of married respondents and 48.7% of unmarried-sexually active respondents were using a modern method of contraception. Of those reporting using a modern method of contraception, implants (38.5%) were the most widely used method by married women, while male condoms (71.6%) were the most widely used method by unmarried-sexually active women. We also observed a higher prevalence of IUCD, injectables and oral contraceptive pill use among married women.

For peer review only

Table 1. Characteristics of married and unmarried-sexually active women aged 15-19 years in Mwanza, Tanzania[1].

Characteristic	Married, N=201	Unmarried, N=744	p value
**Sociodemographic factors**			
**Age (years)**			
15	2 (1.0)	62 (8.3)	
16	4 (2.0)	82 (11.0)	
17	24 (11.9)	161 (21.6)	
18	69 (34.3)	199 (26.8)	
19	102 (50.8)	240 (32.3)	<0.0001
**Age (years)[¥]**	19 (18,19)	18 (17-19)	<0.0001
**Religion**			
Catholic	61 (30.4)	309(41.5)	
Protestant / Other Christian	99 (49.3)	320 (43.0)	
Muslim	38 (18.9)	112 (15.1)	
No religion	3 (1.5)	3 (0.40)	0.04
**Highest level of education achieved**			
No education	15 (7.5)	21 (2.8)	
Primary education	131 (65.2)	320 (43.0)	
Secondary education	55 (27.4)	392 (52.7)	
University education	0	11 (1.5)	<0.0001
**Currently in educational training programme**			
Yes	2 (1.0)	152 (20.4)	
No	199 (99.0)	592 (79.6)	<0.0001
**Socio-economic level**	38 (22.1)		
Lowest Quintile	55 (32.0)	87 (15.5)	
2nd Lowest Quintile	31 (18.0)	131 (23.4)	
Middle Quintile	36 (20.9)	82 (14.6)	
2nd Highest Quintile	12 (7.0)	125 (22.3)	

For peer review only - http://bmjopen.bmj.com/site/about/guidelines.xhtml

Highest Quintile		135 (24.1)	0.0002
**Exposure to information about contraception**			
**Heard about contraception in the media in last 12 months**			
Yes	59 (29.4)	309 (41.5)	
No	142 (70.7)	435 (58.5)	<0.0001
**Heard about contraception from health sector sources in last 12 months**			
Yes	122 (60.7)	213 (28.6)	
No	79 (39.3)	531 (71.4)	<0.0001
**Heard about contraception from interpersonal sources in last 12 months**			
Yes	100 (49.8)	487 (65.5)	
No	101 (50.3)	257 (34.5)	0.0001
**Knows a place where or person from whom she would feel comfortable accessing contraception**			
Yes	113 (61.1)	400 (53.8)	
No	72 (38.9)	343 (46.2)	0.14
**Social networks**			
**Perceives that partner supports her using contraception**			
Yes	116 (62.7)	430 (60.2)	
No	45 (24.3)	140 (19.6)	
Don’t know	24 (13.0)	144 (20.2)	0.04
**Perceives that mother supports her using contraception**			
Yes	89 (50.9)	299 (42.4)	
No	53 (30.3)	190 (26.9)	
Don’t know	33 (18.9)	217 (30.7)	0.03
**Perceives that friends supports her using contraception**			
Yes	85 (46.2)	430 (58.3)	
No	38 (20.7)	100 (13.6)	
Don’t know	61 (33.2)	207 (28.1)	0.02
**Individual knowledge, attitudes and behaviours**			
**Knowledge about contraception score***			
0-1	21 (10.5)	37 (5.0)	
2-3	67 (33.3)	243 (32.7)	
4-5	113 (56.2)	464 (62.4)	0.02
**Misconceptions about contraception score****			
0-1	83 (41.3)	258 (34.7)	
2-3	75 (37.3)	375 (50.4)	
4	43 (21.4)	111 (14.9)	0.04
**Self-efficacy for contraception score*****			
0-1	15 (8.1)	57 (7.7)	
2-3	72 (38.9)	265 (35.7)	
4	98 (53.0)	421 (56.7)	0.67
**Timing of most recent sexual activity**			
Within past week	86 (42.8)	48 (6.5)	
Within past month	52 (25.9)	207 (27.8)	
Within past year	63 (31.3)	489 (65.7)	<0.0001
**Number of living children**			
No children	97 (48.3)	638 (85.8)	
1 child or more	104 (51.7)	106 (14.3)	<0.0001

[1] The figures refers to N (%). Numbers and percentages may not match exactly because the analysis used sampling weights to account for the sampling design.

For peer review only - http://bmjopen.bmj.com/site/about/guidelines.xhtml

¥ Median (Inter quartile range)

* Scored based on the responses to the following five questions: (1) preventing unintended pregnancies is a benefit of contraception, (2) preventing abortions is a benefit of contraception, (3) some contraceptive methods reduce sexually transmitted infections/HIV, (4) modern contraception can help with child spacing, and (5) using modern contraception can allow a woman to complete her education, take up better economic opportunities and fulfil her potential.

** Scored based on the responses to the following four questions: (1) use of a long-acting reversible contraceptive can make adolescent women permanently infertile, (2) changes to normal menstrual bleeding patterns, which is caused by some contraceptives, are harmful to health, (3) modern contraceptives can make adolescent women permanently fat, and (4) adolescent women who use family planning/birth spacing may become promiscuous.

*** Scored based on the responses to the following four questions: (1) felt able to start a conversation with her partner about contraception, (2) felt able to use a method of contraception even if her partner did not want her to, (3) felt able to obtain information on contraception services and products if she needed to, and (4) felt able to obtain a contraceptive method if she decided to use one.

Table 2. Prevalence of contraceptive use among women aged 15–19 years in Mwanza, Tanzania by marital status [1].

Characteristic	Married, N=201[2]	Unmarried, N=744[2]	p value
**Any method**	20.4 (13.9-28.9)	50.7 (47.7-53.6)	<0.0001
**Any modern method[3]**	19.4 (13.4-27.3)	48.7 (45.8-51.5)	
**Any traditional method**	1.0 (0.22-4.4)	2.0 (1.3-3.0)	
**Not currently using**	79.6 (71.2-86.1)	49.3 (46.4-52.3)	<0.0001
Total	100.0	100.0	
**Modern method**			
Implant	38.5 (21.4-58.9)	4.4 (2.4-7.9)	
IUCD	7.7 (2.5-21.5)	0.28 (0.03-2.3)	
Injectables	23.1 (10.8-42.7)	5.8 (3.8-8.7)	
Oral contraceptive pill	2.6 (0.31-18.0)	0.55 (0.13-2.4)	
Emergency pill	0	0.55 (0.12-2.4)	
Male condom	7.7 (2.4-22.2)	71.6 (66.9-75.8)	
Standard Days Method	15.4 (5.2-37.6)	15.8 (12.1-20.3)	
Other modern method	5.1 (1.4-16.6)	1.1 (0.37-3.2)	<0.0001
Total	100.0	100.0	

1 Figures are % (95% confidence intervals) taking into account sampling weights to account for the sampling design.
2 Unmarried girls who report sexually activity in last 12 months; all married girls in Ilemela district.
3 Modern methods include female sterilisation, male sterilisation, contraceptive pill (oral contraceptives), IUCD, injectables (Depo-Provera), implants (Norplant), female condom, male condom, diaphragm, contraceptive foam and contraceptive jelly, LAM, SDM, cycle beads.

In Table 3, we present factors associated with use of modern contraceptive methods among unmarried-sexually active women aged 15 – 19 years in Mwanza, Tanzania.

Socio-demographic factors

For peer review only - http://bmjopen.bmj.com/site/about/guidelines.xhtml

Age, religion, level of education, being in an educational programme and socio-economic position were all associated with use of modern contraceptive methods in univariate analysis (p-value <0.2). The odds of using modern contraception increased with age (adjusted OR (adjOR) 1.2, 95% CI, 1.1-1.4). Following adjustment for age and religion, there was strong evidence that unmarried-sexually active women who had reached university level education were three times more likely to use modern contraception compared to those with no education (adjOR 3.0, 95% CI,1.0, 9.0 , p-value 0.004) and those who were not in education had significantly lower odds of using modern contraception compared to those in education (adjOR 0.52, 95% CI 0.36-0.75).

Exposure to information about contraception

Hearing about contraception in the media in the last 12 months or from an interpersonal source, and knowing of a place or person from whom respondent would feel comfortable accessing contraception were significantly associated with using modern contraception in univariate analysis (p-value < 0.2).

After adjusting for socio-demographic variables, the odds of using modern contraception were significantly lower for unmarried-sexually active women who had not heard about contraception in the media in the last 12 months (adjOR 0.58, 95% CI 0.35-0.95) or from interpersonal sources (adjOR 0.61, 95% CI 0.42-0.90) compared to those that had heard this information.

Social network factors

Perceived support from partner, mother and friends for using contraception were associated with using modern contraception in univariate analysis (p-value <0.2). After adjusting for socio-demographic variables, the odds of using modern contraception were lower for unmarried-sexually active women who perceived that their partners did not support their use of contraception (adjOR

For peer review only - http://bmjopen.bmj.com/site/about/guidelines.xhtml

0.29, 95% CI 0.21-0.42) and similarly for those who perceived lack of support from their friends (adjOR 0.55, 95% CI 0.34-0.88) compared to those who perceived their support.

Individual knowledge, attitudes and behaviour factors

Knowledge about contraception, holding misconceptions against contraception, self-efficacy for contraception and number of living children were associated with using modern contraception in univariate analysis (p-value <0.2). After adjusting for demographic variables, the odds of using modern contraception were significantly lower in unmarried-sexually active women with one or more living children compared to those without a child (adjOR 0.57, 95%CI 0.39-0.85). The odds of using modern contraception increased with higher knowledge about contraception (adjOR 2.4, 95% CI 1.2-4.6) and higher self-efficacy for using contraception (adjOR 2.4, 95% CI 1.5-4.1).

For peer review only

For peer review only - http://bmjopen.bmj.com/site/about/guidelines.xhtml

Table 3. Factors associated with modern contraception use among unmarried sexually active women aged 15–19 years in Mwanza, Tanzania, (N=744)£

Exposure category	No.	Prevalence, n (%)	Unadjusted OR (95% CI)	p value	adjusted OR (95% CI)	p value
**Sociodemographic factors**						
**Age (years)**						
15	62	20 (32.3)				
16	82	30 (36.6)				
17	161	79 (49.1)				
18	199	107 (53.8)				
19	240	126 (52.5)				
Per Year Increase			1.2 (1.1-1.4)	0.01		
**Religion**						
Catholic	309	145 (46.9)	1			
Protestant/Other Christian	320	149 (46.6)	0.99 (0.77-1.3)			
Muslim	112	66 (58.9)	1.6 (1.1-2.4)			
No religion	3	2 (66.7)	2.3 (0.50-10.2)	0.10		
**Highest educational achieved[1]**						
No education	21	7 (33.3)	1		1	
Primary	320	127 (39.7)	1.3 (0.39-4.5)		1.4 (0.40-4.7)	
Secondary	392	221 (56.4)	2.6 (0.83-8.0)	0.0006	2.5 (0.78-8.1)	0.004
University	11	7 (63.6)	3.5 (1.2-9.8)		3.0 (1.0-9.0)	
**Currently in educational training[1]**						
Yes	152	89 (58.6)	1		1	
No	592	273 (46.1)	0.61 (0.43-0.86)	0.008	0.52 (0.36-0.75)	0.002
**Socio-economic level[1]**						
Lowest Quintile	87	36 (41.4)	1		1	
2nd Lowest Quintile	131	62 (47.3)	1.3 (0.71-2.3)		1.2 (0.70-2.1)	
Middle Quintile	82	43 (52.4)	1.6 (0.80-3.0)		1.5 (0.72-3.1)	
2nd Highest Quintile	125	59 (47.2)	1.3 (0.70-2.3)		1.2 (0.60-2.3)	
Highest Quintile	135	79 (58.5)	2.0 (1.1-3.6)	0.08	1.9 (1.1-3.4)	0.09
**Exposure to information about contraception**						
**Heard about contraception in the**						

For peer review only - http://bmjopen.bmj.com/site/about/guidelines.xhtml

**media in last 12months²**						
Yes	309	174 (56.3)	1		1	
No	435	188 (43.2)	0.59 (0.42-0.83)	0.004	0.58 (0.35-0.95)	0.03
**Heard about contraception from health sector sources in last 12months**						
Yes	213	101 (47.4)	1			
No	531	261 (49.2)	1.1 (0.71-1.6)	0.73		
**Heard about contraception from interpersonal sources in last 12months²**						
Yes	487	261 (53.6)	1		1	
No	257	101 (39.3)	0.56 (0.40-0.78)	0.002	0.61 (0.42-0.90)	0.01
**Know of a place where or person from whom she would feel comfortable accessing contraception²**						
Yes	400	213 (53.3)	1		1	
No	343	149 (43.4)	0.67 (0.50-0.92)	0.01	0.69 (0.46-1.0)	0.07
**Social networks**						
**Perceives that partner supports her using contraception²**						
Yes	430	264 (61.4)	1		1	
No	140	40 (28.6)	0.25 (0.18-0.35)		0.29 (0.21-0.42)	
Don't know	144	53 (36.8)	0.37 (0.24-0.55)	<0.0001	0.32 (0.20-0.52)	<0.0001
**Perceives that mother supports her using contraception²**						
Yes	299	160 (53.5)	1		1	
No	190	89 (46.8)	0.77 (0.53-1.1)		0.87 (0.56-1.4)	
Don't know	217	97 (44.7)	0.70 (0.54-0.92)	0.05	0.73 (0.48-1.1)	0.32
**Perceives that friends supports her using contraception²**						
Yes	430	240 (55.8)	1		1	
No	100	44 (44.0)	0.62 (0.45-0.86)		0.55 (0.34-0.88)	
Don't know	207	76 (36.7)	0.46 (0.33-0.63)	0.0004	0.42 (0.29-0.61)	0.0004
**Individual knowledge, attitudes**						

For peer review only - http://bmjopen.bmj.com/site/about/guidelines.xhtml

**and behaviours**						
**Knowledge about**						
**contraception[2,*]**			1		1	
0-1	37	12 (32.4)	1.6 (0.98-2.5)		1.9 (1.0-3.4)	
2-3	243	104 (42.8)	2.4 (1.4-4.0)	0.01	2.4 (1.2-4.6)	0.05
4-5	464	246 (53.0)				
**Misconceptions about**						
**contraception[2,**]**						
0-1	258	114 (44.2)	1		1	
2-3	375	185 (49.3)	1.2 (0.80-1.9)		0.93 (0.58-1.5)	
4	111	63 (56.8)	1.7 (0.96-2.9)	0.19	1.4 (0.82-2.4)	0.34
**Self-efficacy for contraception[2,***]**						
0-2	117	28 (23.9)	1		1	
3-4	626	334 (53.4)	3.6 (2.4-5.5)	<0.0001	2.4 (1.5-4.1)	0.002
**Timing of most recent sexual**						
**activity**						
Within past week	48	24 (50.0)	1			
Within past month	207	110 (53.1)	1.1 (0.58-2.2)			
Within past year	489	228 (46.6)	0.87 (0.47-1.6)	0.42		
**Number of living children[2]**						
No children	638	321 (50.3)	1		1	
1 child or more	106	41 (38.7)	0.62 (0.44-0.89)	0.01	0.57 (0.39-0.85)	0.008

[£] Numbers and percentages may not match exactly because the analysis used sampling weights to account for the sampling design.
OR: odds ratio;
CI: confidence interval; p-value from design based Wald test.

Adjusted ORs: 1 - Adjusted for age and religion. 2 - Adjusted for age, religion, highest education level achieved, currently in education and socioeconomic position.

***** Scored based on the responses to the following five questions: (1) preventing unintended pregnancies is a benefit of contraception, (2) preventing abortions is a benefit of contraception, (3) some contraceptive methods reduce sexually transmitted infections/HIV, (4) modern contraception can help with child spacing, and (5) using modern contraception can allow a woman to complete her education, take up better economic opportunities and fulfil her potential.

****** Scored based on the responses to the following four questions: (1) use of a long-acting reversible contraceptive can make adolescent women permanently infertile, (2) changes to normal menstrual bleeding patterns, which is caused by some contraceptives, are harmful to health, (3) modern contraceptives can make adolescent women permanently fat, and (4) adolescent women who use family planning/birth spacing may become promiscuous.

For peer review only

******* Scored based on the responses to the following four questions: (1) felt able to start a conversation with her partner about contraception, (2) felt able to use a method of contraception even if her partner did not want her to, (3) felt able to obtain information on contraception services and products if she needed to, and (4) felt able to obtain a contraceptive method if she decided to use one.

For peer review only

For peer review only - http://bmjopen.bmj.com/site/about/guidelines.xhtml

DISCUSSION

In this paper, we present socio-demographic and fertility characteristic associated with use of modern contraceptive methods among married and unmarried sexually active women aged 15 to 19 years taking part in the A360 evaluation baseline survey. We also present determinants for modern contraception among unmarried sexually active women aged 15 to 19 years who were enrolled in this study.

For peer review only

We found that more married women (99.0%) than their unmarried counterparts (79.6%) were out of the formal educational system and the proportion of secondary education achievers were 27.4% and 52.7% respectively. Child marriage is prevalent in Tanzania,[14 15] and our findings may highlight the negative impact of child marriage on educational attainment by adolescent women and its attendant effect on the perpetuation of a vicious cycle of poverty at individual, family and community levels.[1 9] However, child marriage is increasingly acknowledged as a violation of girls' human rights which must be protected by family, community and government authorities.[15]

In this study, although both married and unmarried sexually active women showed similar levels of knowledge about contraception (56.2% vs 62.4%) and self-efficacy for modern contraception use (53.0% vs 56.7%), the proportion of modern contraceptive users was far lower among married women when compared to unmarried women (19.4% vs 48.7%). In sub Saharan Africa, modern contraception use has remained low despite the rise of awareness and knowledge.[5] In view of this evidence, our findings could be pointing to presence of other factors that may have a substantial inhibitive role to contraceptive uptake particularly among married adolescent women. One of those determinants is the social pressure for adolescent women to prove their fertility immediately after marriage, [16] which has also been shown to be used to win over husband's respect and stabilise

For peer review only - http://bmjopen.bmj.com/site/about/guidelines.xhtml

marital relationship.[5 17] Such factors may potentially carry more weight and take higher priority over higher knowledge and self efficacy in deciding whether or not to use modern contraception.

The most commonly used contraceptive methods were implants (38.5%) among married women and male condoms (71.6%) among unmarried sexually active women. In a context of held misconceptions particularly against hormonal-based contraceptives for their perceived interference on fertility, this finding might reflect an attempt by unmarried sexually active women to preserve their fertility by opting for non-hormonal based and/or non-invasive methods. Other studies have reported that some adolescent women have chosen unsafe clandestine abortions over hormonal-based contraception.[5 18]. Despite the limitation of condoms being male controlled, the double role of condoms in preventing unintended pregnancies and sexually transmitted infections including HIV, stresses the need for making condoms more accessible and advocating for their proper and regular use among unmarried sexually active adolescent women[7].

Modern contraception use among unmarried sexually active women in the study population was associated with increasing age, increasing levels of education, being in education, hearing of modern contraception from interpersonal sources and in the media in past 12 months, perceiving partner and/or friend support for contraceptive use, as well as higher knowledge about contraception and self efficacy for contraception.

We found that the odds for modern contraception use were low in the respondents who perceived that their partner and/or friends did not approve of their contraception practice compared to those who perceived that their partner and/or friends did approve. Social network support has been consistently shown to influence women's decision to use contraception in various age groups and socio-cultural contexts including in sub-Saharan Africa. [5 19 20] We did not observe an association between perception of mothers support for contraceptive use and use of modern contraceptives

For peer review only - http://bmjopen.bmj.com/site/about/guidelines.xhtml

suggesting that for unmarried adolescent women, partners and friends may be more important influencers than mothers. In addition, we found that, exposure to information about contraception from interpersonal sources or in the media in past 12 months were associated with increased odds for using modern contraception. These findings call for a need for family planning programmes to target the entire community in order to raise awareness of modern contraception and most importantly to engage male to support for the uptake of modern contraception.[20]

Among unmarried sexually active women, higher knowledge and self-efficacy for contraceptive use was associated with increased odds for contraceptive use. This finding, when viewed together with other significant determinants such as advancing age and being in education, underscores the spill-over effect of girls' schooling in delaying early marriage as well as its importance in giving adolescent women more time for mental and physical maturity before embarking on sexual and reproductive roles.[9] Additionally, being in education has a potential role to overcome held misconceptions against modern contraception use.[21] This agrees with another study done in rural Mwanza which had found that low education was a risk factor for unplanned pregnancy in young women. [22]

Among unmarried women, the odds for using modern contraception were found to be significantly lower among those with one or more living children when compared to those without. This being a cross sectional analysis, partly, it could be partly telling that the unmarried women with living children are not contraceptive users in the first place hence risking early pregnancies. But it could also be telling us that unmarried women with living children are more likely to also be young, out of school, with little exposure to information about contraception and low self efficacy to contraception, hence low contraception use. [22 23] In addition, this finding could be pointing to the negative role of mental health issues including depression facing unmarried and/or out of school

For peer review only - http://bmjopen.bmj.com/site/about/guidelines.xhtml

teenage mothers.[23] Despite few studies from low and middle income countries, depression has been shown to be an independent risk factor for repeated teenage pregnancy. [24]

CONCLUSION

In Northwest Tanzania, among married and unmarried sexually active women aged 15 to 19 years, we found strong evidence of key differences in sexuality, fertility and family planning characteristics and modern contraceptive use.

Among unmarried sexually active women, contraceptive use was significantly associated with increasing age, increasing levels of education, being in education, hearing information on modern contraception from interpersonal sources and in the media in the past 12 months, perceiving partner and/or friend support for contraception use, as well as higher knowledge and self efficacy for modern contraception.

In order to optimise their impact, sexual and reproductive health programmes aiming to increase uptake of modern contraceptives should consider the importance of being in education and social support for contraceptive use among adolescent women. Hence the need to focus intervention efforts on more vulnerable unmarried sexually active adolescent women e.g. those with lower education/socioeconomic status and/or those who are already teenage mothers.

CONFLICTING INTERESTS:

The authors declare that they have no competing interests.

FUNDING:

The Bill & Melinda Gates Foundation and the Children's Investment Fund Foundation. The funding bodies had no role in the design of the study and collection, analysis, and interpretation of data and in writing the manuscript.

For peer review only - http://bmjopen.bmj.com/site/about/guidelines.xhtml

AUTHORS' CONTRIBUTIONS:

MKN, CJA, SK, CB and AMD were involved in conception and study design. CB provided statistical expertise. MKN and CJA were involved in drafting of the manuscript. SK, CB and AMD were involved in critical revision of the manuscript for important intellectual content. All the authors were involved in final approval of the manuscript and decision to submit the manuscript for publication.

ACKNOWLEDGEMENTS:
We would like to thank all study participants for their participation and the A360 baseline study team for their dedicated work during data collection.

We also thank Itad as the lead organisation responsible for the overall A360 evaluation. Avenir Health as a partner in the overall A360 evaluation. PSI Headquarters and PSI Tanzania for their support with site selection and engagement in conversation regarding the design.

DATA AVAILABILITY

Individual de-identified data used for this analysis are available from AMD Aoife.Doyle@lshtm.ac.uk on reasonable request.

TABLE LEGEND

Table 1: Characteristics of married and unmarried-sexually active women aged 15-19 years in Mwanza, Tanzania[1].

Table 2: Prevalence of contraceptive use among women aged 15–19 years in Mwanza, Tanzania by marital status [1].

Table 3: Factors associated with modern contraception use among unmarried sexually active women aged 15–19 years in Mwanza, Tanzania, (N=744)[£]

For peer review only - http://bmjopen.bmj.com/site/about/guidelines.xhtml

REFERENCES

1. The World Health Organisation guidelines on preventing early pregnancy and poor reproductive outcomes among adolescents in developing countries. Geneva: WHO, 2011.
2. Blum RW, Gates WH. Girlhood, not motherhood: preventing adolescent pregnnancy. New York: United Nations Populations Fund (UNFPA), 2015.
3. Family planning 2020 initiative [cited 2018 October]. Available from: https://www.familyplanning2020.org/measurement-hub#global-data accessed September 2018.
4. The UN 2030 agenda for sustainable development New York: United Nations; 2015 [cited 2019 Jan]. Available from: https://sustainabledevelopment.un.org/content/documents/21252030%20Agenda%20for%20Sustainable%20Development%20web.pdf accessed October 2018.
5. Blackstone SR, Nwaozuru U, Iwelunmor J. Factors Influencing Contraceptive Use in Sub-Saharan Africa: A Systematic Review. *Int Q Community Health Educ* 2017;37:79-91. doi: 10.1177/0272684X16685254
6. Nalwadda G, Mirembe F, Byamugisha J, et al. Persistent high fertility in Uganda: young people recount obstacles and enabling factors to use of contraceptives. *BMC Public Health* 2010;10:530. doi: 10.1186/1471-2458-10-530
7. Williamson LM, Parkes A, Wight D, et al. Limits to modern contraceptive use among young women in developing countries: a systematic review of qualitative research. *Reprod Health* 2009;6:3. doi: 10.1186/1742-4755-6-3
8. Conde-Agudelo A, Belizan JM, Lammers C. Maternal-perinatal morbidity and mortality associated with adolescent pregnancy in Latin America: Cross-sectional study. *Am J Obstet Gynecol* 2005;192:342-9. doi: 10.1016/j.ajog.2004.10.593
9. Wodon Q, Nayihouba A, Onagoruwa A, et al. Economic impacts of child marriage: global synthesis report. Washington DC: International Centre for Research on Women (ICRW), 2017:43-49.

For peer review only - http://bmjopen.bmj.com/site/about/guidelines.xhtml

10. Atchison CJ, Mulhern E, Kapiga S, et al. Evaluating the impact of an intervention to increase uptake of modern contraceptives among adolescent girls (15 - 19 years) in Nigeria, Ethiopia and Tanzania: the adolescents 360 quasi-experimental study protocol. *BMJ Open* 2018;8:e021834. doi: 021810.021136/bmjopen-022018-021834

11. Ministry of Health (MoHCDGEC) [Tanzania Mainland], Ministry of Health (MoH) [Zanzibar], National Bureau of Statistics (NBS), et al. Tanzania Demographic and Health Survey and Malaria Indicator Survey (TDHS-MIS) 2015-16. Dar es Salaam, Tanzania.: National Bureau of Statistics (NBS), 2016.

12. Central Statistical Agency (CSA) [Ethiopia], and ICF. Ethiopia Demographic and Health Survey 2016. Addis Ababa, Ethiopia and Rockville, Maryland, USA: Central Statistical Agency [Ethiopia], 2016.

13. Tanzania Equity Tool [cited 2019 16 Jan]. Available from: http://www.equitytool.org/tanzania/ accessed 18 Jan 2019.

14. Koski A, Clark S, Nandi A. Has child marriage declined in sub-Saharan Africa? An analysis of trends in 31 countries. *Popul Dev Rev* 2017;43:7 - 29.

15. (Tanzania) MoH. National survey on the drivers and consequences of child marrige in Tanzania. Dar es Salaam - Tanzania: Ministry of Health (MoHCDGEC), 2017.

16. UNDESA. Trends in the contraceptive use worldwide. New York: United Nations Department of Economic and Social Affairs (UNDESA - Population Division). 2015.

17. Eaton L, Flisher AJ, Aar LE. Unsafe sexual behaviour in south African youth. *Soc Sci Med* 2002;56:149 - 65.

18. Plummer ML, Wamoyi J, Nyalali K, et al. Aborting and suspending pregnancy in rural Tanzania: an ethnography of young people's beliefs and practices. *Stud Fam Plann* 2008;39(4):281-92.

19. Omideyi AK, Akinyemi AI, Aina OI, et al. Contraceptive practice, unwanted pregnancies and induced abortion in southwest Nigeria. *Global public health* 2011;6 Suppl 1:S52-72. doi: 10.1080/17441692.2011.594073

For peer review only - http://bmjopen.bmj.com/site/about/guidelines.xhtml

20. Samandari G, Speizer IS, O'Connell K. The role of social support and parity on contraceptive use in Cambodia. *Int Perspect Sex Reprod Health* 2010;36:122-31.

21. Mardi A, Ebadi A, Shahbazi S, et al. Factors influencing the use of contraceptives through the lens of teenage women: a qualitative study in Iran. *BMC Public Health* 2018;18(1):202. doi: 10.1186/s12889-018-5116-3

22. Calvert C, Baisley K, Doyle AM, et al. Risk factors for unplanned pregnancy among young women in Tanzania. *J Fam Plann Reprod Health Care* 2013;39:e2. doi: 10.1136/jfprhc-2012-100389

23. SmithBattle L, Freed P. Teen Mothers' Mental Health. *MCN The American journal of maternal child nursing* 2016;41:31-6; quiz E3-4. doi: 10.1097/NMC.0000000000000198

24. Maravilla JC, Betts KS, e Cruz C, et al. Factors influencing repeated teenage pregnancy: A review and meta analysis. *Am J Obstet Gynecol* 2017;19e

For peer review only

For peer review only - http://bmjopen.bmj.com/site/about/guidelines.xhtml

Reporting checklist for cross sectional study.

Based on the STROBE cross sectional guidelines.

Instructions to authors

Complete this checklist by entering the page numbers from your manuscript where readers will find each of the items listed below.

Your article may not currently address all the items on the checklist. Please modify your text to include the missing information. If you are certain that an item does not apply, please write "n/a" and provide a short explanation.

Upload your completed checklist as an extra file when you submit to a journal.

In your methods section, say that you used the STROBE cross sectional reporting guidelines, and cite them as:

von Elm E, Altman DG, Egger M, Pocock SJ, Gotzsche PC, Vandenbroucke JP. The Strengthening the Reporting of Observational Studies in Epidemiology (STROBE) Statement: guidelines for reporting observational studies.

For peer review only

		Reporting Item	Page Number
Title	#1a	Indicate the study's design with a commonly used term in the title or the abstract	1 & 2
Abstract	#1b	Provide in the abstract an informative and balanced summary of what was done and what was found	2

Background / rationale	#2	Explain the scientific background and rationale for the investigation being reported	4
Objectives	#3	State specific objectives, including any prespecified hypotheses	4
Study design	#4	Present key elements of study design early in the paper	5
Setting	#5	Describe the setting, locations, and relevant dates, including periods of recruitment, exposure, follow-up, and data collection	5
Eligibility criteria	#6a	Give the eligibility criteria, and the sources and methods of selection of participants.	5
	#7	Clearly define all outcomes, exposures, predictors, potential confounders, and effect modifiers. Give diagnostic criteria, if applicable	7 - 9
Data sources / measurement	#8	For each variable of interest give sources of data and details of methods of assessment (measurement). Describe comparability of assessment methods if there is more than one group. Give information separately for exposed and unexposed groups if applicable.	7 - 8
Bias	#9	Describe any efforts to address potential sources of bias	6
Study size	#10	Explain how the study size was arrived at	6
Quantitative variables	#11	Explain how quantitative variables were handled in the analyses. If applicable, describe which groupings were chosen, and why	7 - 9

For peer review only

Statistical methods	#12a	Describe all statistical methods, including those used to control for confounding	8
	#12b	Describe any methods used to examine subgroups and interactions	
	#12c	Explain how missing data were addressed	
	#12d	If applicable, describe analytical methods taking account of sampling strategy	8
	#12e	Describe any sensitivity analyses	
Participants	#13a	Report numbers of individuals at each stage of study—eg numbers potentially eligible, examined for eligibility, confirmed eligible, included in the study, completing follow-up, and analysed. Give information separately for for exposed and unexposed groups if applicable.	10
	#13b	Give reasons for non-participation at each stage	10
	#13c	Consider use of a flow diagram	n/a
Descriptive data	#14a	Give characteristics of study participants (eg demographic, clinical, social) and information on exposures and potential confounders. Give information separately for exposed and unexposed groups if applicable.	10
	#14b	Indicate number of participants with missing data for each variable of interest	10,11,12
Outcome data	#15	Report numbers of outcome events or summary measures.	16 - 18

For peer review only

		Give information separately for exposed and unexposed groups if applicable.	
Main results	#16a	Give unadjusted estimates and, if applicable, confounder-adjusted estimates and their precision (eg, 95% confidence interval). Make clear which confounders were adjusted for and why they were included	16 , 18
	#16b	Report category boundaries when continuous variables were categorized	11 , 12
	#16c	If relevant, consider translating estimates of relative risk into absolute risk for a meaningful time period	n/a
Other analyses	#17	Report other analyses done—e.g., analyses of subgroups and interactions, and sensitivity analyses	n/a
Key results	#18	Summarise key results with reference to study objectives	23
Limitations	#19	Discuss limitations of the study, taking into account sources of potential bias or imprecision. Discuss both direction and magnitude of any potential bias.	3
Interpretation	#20	Give a cautious overall interpretation considering objectives, limitations, multiplicity of analyses, results from similar studies, and other relevant evidence.	23
Generalisability	#21	Discuss the generalisability (external validity) of the study results	3
Funding	#22	Give the source of funding and the role of the funders for the present study and, if applicable, for the original study on which	23

For peer review only

the present article is based

The STROBE checklist is distributed under the terms of the Creative Commons Attribution License CC-BY. This checklist can be completed online using https://www.goodreports.org/, a tool made by the EQUATOR Network in collaboration with Penelope.ai

For peer review only

BMJ Open

Modern contraceptive use among sexually active women aged 15 to 19 years in north-western Tanzania: results from the Adolescent 360 (A360) baseline survey

Journal:	*BMJ Open*
Manuscript ID	bmjopen-2019-030485.R1
Article Type:	Research
Date Submitted by the Author:	29-May-2019
Complete List of Authors:	Nsanya, Mussa; Mwanza Intervention Trials Unit, Atchison, Christina; London School of Hygiene and Tropical Medicine Faculty of Epidemiology and Population Health, MRC Tropical Epidemiology Group Bottomley, Christian ; London School of Hygiene and Tropical Medicine Faculty of Epidemiology and Population Health, MRC Tropical Epidemiology Group Doyle, Aoife; London School of Hygiene and Tropical Medicine Faculty of Epidemiology and Population Health, MRC Tropical Epidemiology Group Kapiga, Saidi ; London School of Hygiene and Tropical Medicine, Infectious Disease Epidemiology; London School of Hygiene and Tropical Medicine Faculty of Epidemiology and Population Health, MRC Tropical Epidemiology Group
**Primary Subject Heading**:	Public health
Secondary Subject Heading:	Reproductive medicine
Keywords:	Adolescents, Contraception, Family Planning, Reproductive health, Africa

SCHOLARONE™ Manuscripts

TITLE

Modern contraceptive use among sexually active women aged 15 to 19 years in north-western Tanzania: results from the Adolescent 360 (A360) baseline survey

Key words: Adolescents, Contraception, Family Planning, Reproductive health, Africa

Authors:

Mussa Kelvin Nsanya[1*] , Christina J. Atchison[2], Christian Bottomley[2], Aoife M. Doyle[2], and Saidi H. Kapiga[1,3]

Institutional Affiliations:

1.Mwanza Intervention Trials Unit/National Institute for Medical Research, P.O. Box 11936, Mwanza, Tanzania

2. MRC Tropical Epidemiology Group, London School of Hygiene and Tropical Medicine, Keppel Street, London WC1E 7H, UK

3. Department of Infectious Disease Epidemiology, London School of Hygiene and Tropical Medicine, Keppel Street, London WC1E 7H, UK

*Corresponding author: mussa.nsanya@mitu.or.tz / kelvin.nsanya@gmail.com

For peer review only

ABSTRACT

Objectives: To describe differences in modern contraceptive use among adolescent women aged 15 to 19 years according to their marital status and to determine factors associated with modern contraceptive use among sexually active women in this population.

Design: Cross-sectional analysis of Adolescent 360 (A360) evaluation baseline survey.

Setting: The 15 urban and semi-urban wards of Ilemela district, Mwanza region, North-western Tanzania.

Participants: Adolescent women aged 15 – 19 years who were living in the study site from August 2017 to February 2018 and who provided informed consent. Women were classified as married if they had a husband or were living as married. Unmarried women were classified as sexually active if they reported having sexual intercourse in the past 12 months.

Outcome measure: Prevalence of modern contraceptive (mCPR) among adolescent women aged 15 – 19 years.

Results: Data was available for 3,511 women aged 15-19 years, of which 201(5.7%) were married and 744 (22.5%) were unmarried-sexually active. We found strong evidence of differences in use of modern contraceptive methods according to marital status of adolescent women. Determinants of modern contraception use among unmarried-sexually active women were increasing age, increasing level of education, being in education, hearing of modern contraception from interpersonal sources or in the media in the past 12 months, perceiving partner and/or friends support for contraceptive use, as well as higher knowledge and self efficacy for contraception.

Conclusions: Sexual and reproductive health programmes aiming to increase uptake of modern contraceptives in this population of adolescent women should consider the importance of girl's education and social support for contraceptive use particularly among unmarried sexually active women.

For peer review only - http://bmjopen.bmj.com/site/about/guidelines.xhtml

Strengths and limitations of this study

- We focused on adolescent women aged 15 – 19 years, a population that is often excluded or underrepresented in most of the studies on modern contraception.
- We used the probability sampling approach to interview 3,511 adolescent women from 34 streets in the 15 urban and semi-urban wards of Ilemela district, Mwanza. Therefore, while it may not be possible to generalize our findings to the wider population of adolescent women aged 15 to 19 years living in similar urban and semi-urban wards of other regions in Tanzania, the sampling approach used allows us to generalise our findings to the wider population of adolescent women aged 15 to 19 years living in urban and semi-urban wards of Ilemela district.
- The main limitations are: the cross-sectional design makes temporal causal relationships hard to establish and reverse causality is likely, for example, use of contraceptives may lead to higher knowledge about contraceptives.
- We had relatively low response rate (68.6%) of potentially eligible women, mainly due to young women being at school, even after three visits in attempt to hold interviews.
- Also, because of the small sample size of married women, the study had limited power to identify determinants for contraceptive use in this group and hence the decision to drop them from the regression analysis.
- We did not specifically ask the adolescent women whether they were planning on getting pregnant shortly, hence we have no data on this potential explanatory variable. In addition, pregnant women were not asked about contraception as they were not "at risk of pregnancy", the same applied to those in post-partum amenorrhea. So we don't have outcome data for these two subgroups. These are fertility characteristic variables that could make a difference primarily between married and unmarried adolescent girls.

For peer review only

For peer review only - http://bmjopen.bmj.com/site/about/guidelines.xhtml

INTRODUCTION

Globally, approximately 16 million women aged 15 to 19 years give birth each year and 95% of these births take place in low and middle income countries.[1 2] The global community, through the Family Planning 2020 (FP2020) initiative, is committed to increase new contraceptive users to 120 million in 69 developing countries (including Tanzania) by 2020. [3] This initiative would also support the objectives of the United Nation's Sustainable Development Goal (SDG) 3 on health and wellbeing for all and SDG 5 on gender equality which also embodies sexual and reproductive health at the heart of global efforts to sustainable development particularly in low and middle income countries.[4]

Modern contraceptive use remains unacceptably low in sub-Saharan Africa despite increasing awareness and knowledge about contraception.[5 6] For instance, 69% and 59% of the women aged 15 to 19 years in United Kingdom and United States of America respectively report using a modern contraceptive at the last time they had sexual intercourse compared to 12% in Mali and 21% in Tanzania.[7] The low uptake of modern contraceptives particularly among women aged 15 to 19 years contributes significantly to high rates of adolescent pregnancies and poor health outcomes including maternal morbidity and mortality, and neonatal and under-five child mortality.[1 5 8] In addition, there are other severe social and economic consequences to adolescent women, their families and whole society including not reaching their potential for educational achievement, and not getting a paid job which usually leads into a vicious cycle of poverty. [1 9]

Most studies to date have focused on the factors that prevent women of reproductive age (15 – 49 years) from using modern contraceptives.[5] In such studies adolescent women are usually underrepresented despite facing disproportionate medical, social and economic impact of

For peer review only - http://bmjopen.bmj.com/site/about/guidelines.xhtml

unintended pregnancies.[1] In order for the goals of FP2020 and SDG 3 and 5 to be achieved, more information is required from studies which examine factors associated with contraceptive use among adolescent women particulary in developing countries, including Tanzania. Moreover, demand for and access to modern contraceptives among adolescent women aged 15 to 19 years are known to differ with the women's marital status. [10] It is therefore important to describe differences according to marital status to optimise access and use of modern contraception.

Adolescents 360 (A360) is an initiative being rolled out across Ethiopia, Nigeria and Tanzania, aiming to increase uptake of voluntary modern contraception among sexually active women aged 15 to 19 years.[11] Using baseline survey data collected as part of A360 programme evaluation, we describe differences in modern contraceptive use among adolescent women aged 15 to 19 years according to their marital status; and determine factors associated with modern contraceptive use among unmarried sexually active women in this population in Mwanza, Tanzania.

METHODS

Study design and settings

Between August 2017 and February 2018, we conducted a cross-sectional baseline survey among women aged 15 to 19 years in Mwanza city, Tanzania. The survey was part of a comprehensive outcome evaluation to assess the impact of the A360 programme on a number of sexual and reproductive health outcomes, primarily uptake of voluntary modern contraception among sexually active women aged 15 to 19 years.[11]

In Tanzania, A360 is being implemented in 16 administrative regions. This survey was conducted in fifteen urban and semi-urban wards of Ilemela district, Mwanza region.[11] Ilemela district covers the northern part of Mwanza city and is comprised of 19 wards, of which 4 are rural wards, 6 are semi-

For peer review only - http://bmjopen.bmj.com/site/about/guidelines.xhtml

urban and 9 are urban wards. Each ward is administratively divided into a number of neighbourhoods, called 'streets'.

Study population

Women were included in the study if they were 15 to 19 years old; living in the study sites at the time of the survey; and voluntarily provided informed consent. Women were classified as married if they reported that they had a husband or were living as married with a cohabiting male partner.

Informed consent

Written informed consent was obtained from all participants. A parental consent waiver was granted for unmarried women aged 15 to 17 years, given the sensitive nature of the topics discussed. Married women under 18 years of age were considered emancipated and did not require parental consent in addition to their own voluntary consent.

Ethics approval

The study was approved by the Tanzania National Health Research Ethics review sub-committee of the National Institute for Medical Research (Ref: NIMR/HQ/R8a/Vol.IX/2549), and the research ethics committee of the London School of Hygiene & Tropical Medicine (LSHTM, Ref: 14145).

Sampling strategy and sample size

A cluster sampling design was used. The primary sampling unit (PSU) for the survey was a 'street', the smallest administrative unit similar to a neighbourhood or a localised and delineated group of people. All 15 urban or semi-urban wards of Ilemela district were included in the survey. Each ward has an estimated eight to ten streets. A simple random sample of 34 'streets' was selected across the 15 urban and semi-urban wards of Ilemela district. As per study protocol, in the first eight 'streets', we randomly selected 50 GPS coordinates using ArcGIS software version 9.3 (Esri,

For peer review only - http://bmjopen.bmj.com/site/about/guidelines.xhtml

Redlands, USA). All households whose front door was located within a radius of 20 meters around the GPS coordinates were visited and all eligible consenting women aged 15 to 19 years residing in these households were invited to be interviewed. This approach was considered due to lack of detailed lists of households in the streets which could serve as a sampling frame. [12] As fewer eligible women than predicted were surveyed in each cluster using this sampling strategy, in the remaining 26 ‘streets’ we visited all households and administered the questionnaire to all eligible and consenting women aged 15 to 19 years. Our target sample size for the baseline survey was 3,314 women aged 15 to 19 years. Sample size and power calculations were conducted ahead of deciding on the number of streets to sample. The estimated sample size had 90% power to detect a 6% increase in prevalence of modern contraceptive use in presence of A360 intervention for 24 months. [11]

If potentially eligible participants were not available at the first visit, two further revisits were made to attempt to hold interviews.

Participants and Public Involvement

We sought permission from local government authorities in the wards where the study took place as well as from individual participants prior to enrollment. Additionally, we have communicated the A360 baseline survey report to local government officials in Ilemela district, Mwanza

Tool for baseline survey

The questionnaire was adapted from various research instruments that have been used in the target countries including the Tanzania Demographic and Health Survey (DHS)[13] and FP2020 survey instruments[3]. Questionnaires were administered face-to-face by female interviewers aged between 18 and 26 years using pre-programmed tablet computers.[11]

For peer review only - http://bmjopen.bmj.com/site/about/guidelines.xhtml

The questionnaire had three components: (1) socio-demographic characteristics, (2) fertility characteristics and preferences, and (3) contraceptive knowledge, attitudes and practices. Only respondents who reported sexual intercourse in the last 12 months were considered sexually active hence asked questions about contraceptive use.[11]

Study outcome

The prevalence of modern contraceptives (mCPR) among married women aged 15 to 19 years was defined as per the DHS definition[14]:

$$\frac{\text{Number of married 15–19-year-old women reporting use of modern contraceptives at the time of the survey}}{\text{Number of married 15–19-year-old women}}$$

mCPR among unmarried-sexually active women aged 15 to 19 years was defined as follows:

$$\frac{\text{Number of unmarried-sexually active* women aged 15–19-year-old reporting use of modern contraceptives at the time of the survey}}{\text{Number of unmarried-sexually active* unmarried women aged 15–19-year-old}}$$

*self-reported that they were sexually active in the 12 months prior to the survey

Modern contraception was defined to include the following: male and female sterilisation, contraceptive implants, intrauterine contraceptive devices (IUCD), injectables, oral contraceptive

For peer review only - http://bmjopen.bmj.com/site/about/guidelines.xhtml

pill, emergency contraceptive pill, male condom, female condom, Standard Days Method (SDM), Lactational Amenorrhoea Method (LAM), diaphragm, spermicides, foams and jelly.[15]

Statistical analysis

Data analysis was conducted in Stata 15. We used sampling weights and robust standard errors to account for the clustered sampling design.

Descriptive data analysis was done for both married and unmarried women. Logistic regression was performed for unmarried-sexually active women only due to small sample size for married women. We obtained odds ratios (OR) for the association of each explanatory variable with use of modern contraception. Wald tests adjusted for the clustered sampling design were used at each step of the analysis. The associations between mCPR and age and between mCPR and religion were not adjusted for other explanatory variables. Age and religion were considered a priori potential confounders for the associations between mCPR and highest education level achieved, currently being in education, type of area of residence (urban or semi-urban) and socioeconomic position. The remaining explanatory variables with p-value < 0.2 in the univariate analysis, were investigated one-by-one in multivariate regression models adjusted for age, religion, highest education level achieved, currently being in education and socioeconomic position. Variables with p value < 0.05 in the adjusted analysis were considered to be associated with mCPR. This strategy allowed us to assess the effect of variables adjusted for distal a priori potential confounders.

Socioeconomic position was created from a series of questions about household items, dwelling materials and access to a bank account. The variable was generated using the "Tanzania Equity Tool" which uses different weights attached to each answer to create a composite score which was then split into quintiles according to the national thresholds.[16]

For peer review only

Knowledge about contraception was assessed through the respondents affirmative report to the following five questions: (1) preventing unintended pregnancies is a benefit of contraception, (2) preventing abortions is a benefit of contraception, (3) some contraceptive methods reduce sexually transmitted infections/HIV, (4) modern contraception can help with child spacing, and (5) using modern contraception can allow a woman to complete her education, take up better economic opportunities and fulfil her potential.

Holding misconceptions was assessed by asking respondents whether they agreed with the following four statements: (1) use of a long-acting reversible contraceptive can make adolescent women permanently infertile, (2) changes to normal menstrual bleeding patterns, which is caused by some contraceptives, are harmful to health, (3) modern contraceptives can make adolescent women permanently fat, and (4) adolescent women who use family planning/birth spacing may become promiscuous.

Self-efficacy for contraception was assessed through four questions relating to the woman's ability to access and use contraception: (1) felt able to start a conversation with her partner about contraception, (2) felt able to use a method of contraception even if her partner did not want her to, (3) felt able to obtain information on contraception services and products if she needed to, and (4) felt able to obtain a contraceptive method if she decided to use one.

Variables for contraception knowledge, holding misconceptions, and self-efficacy were created as scores from 0 - 5 for knowledge, and 0 - 4 for holding misconceptions and self-efficacy based on the overall score for each individual statement in each category. A score of 1 was given if the respondent agreed with the statement and 0 if she disagreed or answered "*don't know*". A maximum score of 5 for knowledge and 4 for self-efficacy would indicate that the respondent correctly agreed with all five knowledge statements and felt able to achieve all four self-efficacy behaviours. A maximum

For peer review only - http://bmjopen.bmj.com/site/about/guidelines.xhtml

score of 4 for holding misconceptions would be interpreted as believing all four myth statements about contraception.

RESULTS

A total of 14,138 households were identified and 99.6% were interviewed to obtain information on household members. A total of 5,121 potentially eligible women aged 15–19 years were identified from 3,963 households (28.1% of all interviewed households); 68.6% (3,511) of potentially eligible women were interviewed, of whom 5.7% (201/3,511) were married. Overall, 22.5% (744/3,310) of unmarried women had been sexually active during the 12 months preceding the survey. The most common reason for a potentially eligible woman not interviewed was being absent or unavailable at home after a maximum of three visits mainly due to attending school.

In Table 1, we present the characteristic of married and unmarried-sexually active women aged 15 to 19 years in the study population. The median age was 19 years for married women and 18 years for unmarried-sexually active women. The majority of respondents were Christian (married: 79.7%, unmarried: 84.5%) and were not currently pursuing educational training (married: 99.0%, unmarried: 79.6%). The highest level of education achieved by majority of married women was primary education (65.2%) while the majority of unmarried-sexually active women had achieved secondary level education (52.7%). Most respondents had moderate knowledge about contraception (married: 56.2%, unmarried: 62.4%) and a moderate self-efficacy for contraception (married: 53.0%, unmarried: 56.7%).

In Table 2, we present the prevalence of contraceptive use among women aged 15 to 19 years in Mwanza by their marital status. Overall, 19.4% of married respondents and 48.7% of unmarried-sexually active respondents were using a modern method of contraception. Of those reporting using a modern method of contraception, implants (38.5%) were the most widely used method by married

For peer review only - http://bmjopen.bmj.com/site/about/guidelines.xhtml

women, followed by injectables (23.1%) and Standard Days Method (15.4%). Male condoms (71.6%) were the most widely used modern contraceptive method by unmarried-sexually active women, followed by Standard Days Method (15.8%). Overall, there was strong evidence of differences in use of modern contraceptive methods according to marital status of adolescent women (Table 2).

For peer review only

Table 1. Characteristics of married and unmarried-sexually active women aged 15-19 years in Mwanza, Tanzania[1].

Characteristic	Married, N=201	Unmarried, N=744	p value
**Sociodemographic factors**	N (%)	N (%)	
**Age (years)**			
15	2 (1.0)	62 (8.3)	
16	4 (2.0)	82 (11.0)	
17	24 (11.9)	161 (21.6)	
18	69 (34.3)	199 (26.8)	
19	102 (50.8)	240 (32.3)	<0.0001
**Age (years)¥**	19 (18,19)	18 (17-19)	<0.0001
**Religion**			
Catholic	61 (30.4)	309(41.5)	
Protestant / Other Christian	99 (49.3)	320 (43.0)	
Muslim	38 (18.9)	112 (15.1)	
No religion	3 (1.5)	3 (0.40)	0.04
**Highest level of education achieved**			
No education	15 (7.5)	21 (2.8)	
Primary education	131 (65.2)	320 (43.0)	
Secondary education	55 (27.4)	392 (52.7)	
University education	0	11 (1.5)	<0.0001
**Currently in educational training programme**			
Yes	2 (1.0)	152 (20.4)	
No	199 (99.0)	592 (79.6)	<0.0001
**Type of area of residence**			
Semi-urban	85 (42.3)	290 (39.0)	
Urban	116 (57.7)	454 (61.0)	0.43
**Socio-economic level**			
Lowest Quintile	38 (22.1)	87 (15.5)	
2nd Lowest Quintile	55 (32.0)	131 (23.4)	
Middle Quintile	31 (18.0)	82 (14.6)	
2nd Highest Quintile	36 (20.9)	125 (22.3)	
Highest Quintile	12 (7.0)	135 (24.1)	0.0002
**Exposure to information about contraception**			
**Heard about contraception in the media in last 12 months**	59 (29.4)		
Yes	142 (70.7)	309 (41.5)	

For peer review only - http://bmjopen.bmj.com/site/about/guidelines.xhtml

No		435 (58.5)	<0.0001
**Heard about contraception from health sector sources in last 12 months**			
Yes	122 (60.7)	213 (28.6)	
No	79 (39.3)	531 (71.4)	<0.0001
**Heard about contraception from interpersonal sources in last 12 months**			
Yes	100 (49.8)	487 (65.5)	
No	101 (50.3)	257 (34.5)	0.0001
**Knows a place where or person from whom she would feel comfortable accessing contraception**			
Yes	113 (61.1)	400 (53.8)	
No	72 (38.9)	343 (46.2)	0.14
**Social networks**			
**Perceives that partner supports her using contraception**			
Yes	116 (62.7)	430 (60.2)	
No	45 (24.3)	140 (19.6)	
Don't know	24 (13.0)	144 (20.2)	0.04
**Perceives that mother supports her using contraception**			
Yes	89 (50.9)	299 (42.4)	
No	53 (30.3)	190 (26.9)	
Don't know	33 (18.9)	217 (30.7)	0.03
**Perceives that friends supports her using contraception**			
Yes	85 (46.2)	430 (58.3)	
No	38 (20.7)	100 (13.6)	
Don't know	61 (33.2)	207 (28.1)	0.02
**Individual knowledge, attitudes and behaviours**			
**Knowledge about contraception score***			
0-1	21 (10.5)	37 (5.0)	
2-3	67 (33.3)	243 (32.7)	
4-5	113 (56.2)	464 (62.4)	0.02
**Misconceptions about contraception score****			
0-1	83 (41.3)	258 (34.7)	
2-3	75 (37.3)	375 (50.4)	
4	43 (21.4)	111 (14.9)	0.04
**Self-efficacy for contraception score*****			
0-1	15 (8.1)	57 (7.7)	
2-3	72 (38.9)	265 (35.7)	
4	98 (53.0)	421 (56.7)	0.67
**Timing of most recent sexual activity**			
Within past week	86 (42.8)	48 (6.5)	
Within past month	52 (25.9)	207 (27.8)	
Within past year	63 (31.3)	489 (65.7)	<0.0001
**Number of living children**			
No children	97 (48.3)	638 (85.8)	
1 child or more	104 (51.7)	106 (14.3)	<0.0001

[1] The figures refers to N (%). Numbers and percentages may not match exactly because the analysis used sampling weights to account for the sampling design.

[¥] Median (Inter quartile range)

***** Scored based on the responses to the following five questions: (1) preventing unintended pregnancies is a benefit of contraception, (2) preventing abortions is a benefit of contraception, (3) some contraceptive methods reduce sexually

For peer review only - http://bmjopen.bmj.com/site/about/guidelines.xhtml

transmitted infections/HIV, (4) modern contraception can help with child spacing, and (5) using modern contraception can allow a woman to complete her education, take up better economic opportunities and fulfil her potential.

** Scored based on the responses to the following four questions: (1) use of a long-acting reversible contraceptive can make adolescent women permanently infertile, (2) changes to normal menstrual bleeding patterns, which is caused by some contraceptives, are harmful to health, (3) modern contraceptives can make adolescent women permanently fat, and (4) adolescent women who use family planning/birth spacing may become promiscuous.

*** Scored based on the responses to the following four questions: (1) felt able to start a conversation with her partner about contraception, (2) felt able to use a method of contraception even if her partner did not want her to, (3) felt able to obtain information on contraception services and products if she needed to, and (4) felt able to obtain a contraceptive method if she decided to use one.

Table 2. Prevalence of contraceptive use among women aged 15–19 years in Mwanza, Tanzania by marital status [1].

Characteristic	Married, N=201[2]	Unmarried, N=744[2]	p value
**Any method**	20.4 (13.9-28.9)	50.7 (47.7-53.6)	<0.0001
**Any modern method[3]**	19.4 (13.4-27.3)	48.7 (45.8-51.5)	
**Any traditional method**	1.0 (0.22-4.4)	2.0 (1.3-3.0)	
**Not currently using**	79.6 (71.2-86.1)	49.3 (46.4-52.3)	<0.0001
Total	100.0	100.0	
**Modern method**			
Implant	38.5 (21.4-58.9)	4.4 (2.4-7.9)	
IUCD	7.7 (2.5-21.5)	0.28 (0.03-2.3)	
Injectables	23.1 (10.8-42.7)	5.8 (3.8-8.7)	
Oral contraceptive pill	2.6 (0.31-18.0)	0.55 (0.13-2.4)	
Emergency pill	0	0.55 (0.12-2.4)	
Male condom	7.7 (2.4-22.2)	71.6 (66.9-75.8)	
Standard Days Method	15.4 (5.2-37.6)	15.8 (12.1-20.3)	
Other modern method	5.1 (1.4-16.6)	1.1 (0.37-3.2)	<0.0001
Total	100.0	100.0	

1 Figures are % (95% confidence intervals) taking into account sampling weights to account for the sampling design.
2 Unmarried girls who report sexually activity in last 12 months; all married girls in Ilemela district.
3 Modern methods include female sterilisation, male sterilisation, contraceptive pill (oral contraceptives), IUCD, injectables (Depo-Provera), implants (Norplant), female condom, male condom, diaphragm, contraceptive foam and contraceptive jelly, LAM, SDM, cycle beads.

In Table 3, we present factors associated with use of modern contraceptive methods among unmarried-sexually active women aged 15 – 19 years in Mwanza, Tanzania.

Socio-demographic factors

Age, religion, level of education, being in an educational programme and socio-economic position were all associated with use of modern contraceptive methods in univariate analysis (p-value <0.2).

For peer review only - http://bmjopen.bmj.com/site/about/guidelines.xhtml

The odds of using modern contraception increased with age (adjusted OR (adjOR) 1.2, 95% CI, 1.1-1.4). Following adjustment for age and religion, there was strong evidence that unmarried-sexually active women who had reached university level education were three times more likely to use modern contraception compared to those with no education (adjOR 3.0, 95% CI,1.0, 9.0 , p-value 0.004) and those who were not in education had significantly lower odds of using modern contraception compared to those in education (adjOR 0.52, 95% CI 0.36-0.75).

Exposure to information about contraception

Hearing about contraception in the media in the last 12 months or from an interpersonal source, and knowing of a place or person from whom respondent would feel comfortable accessing contraception were significantly associated with using modern contraception in univariate analysis (p-value < 0.2).

After adjusting for socio-demographic variables, the odds of using modern contraception were significantly lower for unmarried-sexually active women who had not heard about contraception in the media in the last 12 months (adjOR 0.58, 95% CI 0.35-0.95) or from interpersonal sources (adjOR 0.61, 95% CI 0.42-0.90) compared to those that had heard this information.

Social network factors

Perceived support from partner, mother and friends for using contraception were associated with using modern contraception in univariate analysis (p-value <0.2). After adjusting for socio-demographic variables, the odds of using modern contraception were lower for unmarried-sexually active women who perceived that their partners did not support their use of contraception (adjOR 0.29, 95% CI 0.21-0.42) and similarly for those who perceived lack of support from their friends (adjOR 0.55, 95% CI 0.34-0.88) compared to those who perceived their support.

For peer review only

For peer review only - http://bmjopen.bmj.com/site/about/guidelines.xhtml

Individual knowledge, attitudes and behaviour factors

Knowledge about contraception, holding misconceptions against contraception, self-efficacy for contraception and number of living children were associated with using modern contraception in univariate analysis (p-value <0.2). After adjusting for demographic variables, the odds of using modern contraception were significantly lower in unmarried-sexually active women with one or more living children compared to those without a child (adjOR 0.57, 95%CI 0.39-0.85). The odds of using modern contraception increased with higher knowledge about contraception (adjOR 2.4, 95% CI 1.2-4.6) and higher self-efficacy for using contraception (adjOR 2.4, 95% CI 1.5-4.1).

For peer review only

Table 3. Factors associated with modern contraception use among unmarried sexually active women aged 15–19 years in Mwanza, Tanzania, (N=744)£

Exposure category	No.	Prevalence, n (%)	Unadjusted OR (95% CI)	p value	adjusted OR (95% CI)	p value
**Sociodemographic factors**						
**Age (years)**						
15	62	20 (32.3)				
16	82	30 (36.6)				
17	161	79 (49.1)				
18	199	107 (53.8)				
19	240	126 (52.5)				
Per Year Increase			1.2 (1.1-1.4)	0.01		
**Religion**						
Catholic	309	145 (46.9)	1			
Protestant/Other Christian	320	149 (46.6)	0.99 (0.77-1.3)			
Muslim	112	66 (58.9)	1.6 (1.1-2.4)			
No religion	3	2 (66.7)	2.3 (0.50-10.2)	0.10		
**Highest educational achieved[1]**						
No education	21	7 (33.3)	1		1	
Primary	320	127 (39.7)	1.3 (0.39-4.5)		1.4 (0.40-4.7)	
Secondary	392	221 (56.4)	2.6 (0.83-8.0)		2.5 (0.78-8.1)	
University	11	7 (63.6)	3.5 (1.2-9.8)	0.0006	3.0 (1.0-9.0)	0.004
**Currently in educational training[1]**						
Yes	152	89 (58.6)	1		1	
No	592	273 (46.1)	0.61 (0.43-0.86)	0.008	0.52 (0.36-0.75)	0.002
**Type of area of residence**						
Semi-urban	290	140 (48.3)	1			
Urban	454	222 (48.9)	1.0 (0.82-1.3)	0.82		
**Socio-economic level[1]**						
Lowest Quintile	87	36 (41.4)	1		1	
2nd Lowest Quintile	131	62 (47.3)	1.3 (0.71-2.3)		1.2 (0.70-2.1)	
Middle Quintile	82	43 (52.4)	1.6 (0.80-3.0)		1.5 (0.72-3.1)	
2nd Highest Quintile	125	59 (47.2)	1.3 (0.70-2.3)		1.2 (0.60-2.3)	
Highest Quintile	135	79 (58.5)	2.0 (1.1-3.6)	0.08	1.9 (1.1-3.4)	0.09
**Exposure to information about**						

For peer review only - http://bmjopen.bmj.com/site/about/guidelines.xhtml

**contraception**						
**Heard about contraception in the media in last 12months[2]**						
Yes	309	174 (56.3)	1		1	
No	435	188 (43.2)	0.59 (0.42-0.83)	0.004	0.58 (0.35-0.95)	0.03
**Heard about contraception from health sector sources in last 12months**						
Yes	213	101 (47.4)	1			
No	531	261 (49.2)	1.1 (0.71-1.6)	0.73		
**Heard about contraception from interpersonal sources in last 12months[2]**						
Yes	487	261 (53.6)	1		1	
No	257	101 (39.3)	0.56 (0.40-0.78)	0.002	0.61 (0.42-0.90)	0.01
**Know of a place where or person from whom she would feel comfortable accessing contraception[2]**						
Yes	400	213 (53.3)	1		1	
No	343	149 (43.4)	0.67 (0.50-0.92)	0.01	0.69 (0.46-1.0)	0.07
**Social networks**						
**Perceives that partner supports her using contraception[2]**						
Yes	430	264 (61.4)	1		1	
No	140	40 (28.6)	0.25 (0.18-0.35)		0.29 (0.21-0.42)	
Don't know	144	53 (36.8)	0.37 (0.24-0.55)	<0.0001	0.32 (0.20-0.52)	<0.0001
**Perceives that mother supports her using contraception[2]**						
Yes	299	160 (53.5)	1		1	
No	190	89 (46.8)	0.77 (0.53-1.1)		0.87 (0.56-1.4)	
Don't know	217	97 (44.7)	0.70 (0.54-0.92)	0.05	0.73 (0.48-1.1)	0.32
**Perceives that friends supports her using contraception[2]**	430	240 (55.8)				
Yes	100	44 (44.0)	1		1	
No	207	76 (36.7)	0.62 (0.45-0.86)		0.55 (0.34-0.88)	

For peer review only - http://bmjopen.bmj.com/site/about/guidelines.xhtml

Don't know			0.46 (0.33-0.63)	0.0004	0.42 (0.29-0.61)	0.0004
**Individual knowledge, attitudes and behaviours**						
**Knowledge about contraception[2,*]**			1		1	
0-1	37	12 (32.4)	1.6 (0.98-2.5)		1.9 (1.0-3.4)	
2-3	243	104 (42.8)	2.4 (1.4-4.0)	0.01	2.4 (1.2-4.6)	0.05
4-5	464	246 (53.0)				
**Misconceptions about contraception[2,**]**						
0-1	258	114 (44.2)	1		1	
2-3	375	185 (49.3)	1.2 (0.80-1.9)		0.93 (0.58-1.5)	
4	111	63 (56.8)	1.7 (0.96-2.9)	0.19	1.4 (0.82-2.4)	0.34
**Self-efficacy for contraception[2,***]**						
0-2	117	28 (23.9)	1		1	
3-4	626	334 (53.4)	3.6 (2.4-5.5)	<0.0001	2.4 (1.5-4.1)	0.002
**Timing of most recent sexual activity**						
Within past week	48	24 (50.0)	1			
Within past month	207	110 (53.1)	1.1 (0.58-2.2)			
Within past year	489	228 (46.6)	0.87 (0.47-1.6)	0.42		
**Number of living children[2]**						
No children	638	321 (50.3)	1		1	
1 child or more	106	41 (38.7)	0.62 (0.44-0.89)	0.01	0.57 (0.39-0.85)	0.008

£ Numbers and percentages may not match exactly because the analysis used sampling weights to account for the sampling design.
OR: odds ratio;
CI: confidence interval; p-value from design based Wald test.

Adjusted ORs: 1 - Adjusted for age and religion. 2 - Adjusted for age, religion, highest education level achieved, currently in education and socioeconomic position.

***** Scored based on the responses to the following five questions: (1) preventing unintended pregnancies is a benefit of contraception, (2) preventing abortions is a benefit of contraception, (3) some contraceptive methods reduce sexually transmitted infections/HIV, (4) modern contraception can help with child spacing, and (5) using modern contraception can allow a woman to complete her education, take up better economic opportunities and fulfil her potential.

****** Scored based on the responses to the following four questions: (1) use of a long-acting reversible contraceptive can make adolescent women permanently infertile, (2) changes to normal menstrual bleeding patterns, which is caused by some contraceptives, are harmful to health, (3) modern contraceptives can make adolescent women permanently fat, and (4) adolescent women who use family planning/birth spacing may become promiscuous.

For peer review only - http://bmjopen.bmj.com/site/about/guidelines.xhtml

******* Scored based on the responses to the following four questions: (1) felt able to start a conversation with her partner about contraception, (2) felt able to use a method of contraception even if her partner did not want her to, (3) felt able to obtain information on contraception services and products if she needed to, and (4) felt able to obtain a contraceptive method if she decided to use one.

For peer review only

For peer review only - http://bmjopen.bmj.com/site/about/guidelines.xhtml

DISCUSSION

In this paper, we describe differences in modern contraceptive use among adolescecnt women aged 15 to 19 years taking part in the A360 evaluation baseline survey according to their marital status. We also present determinants for modern contraception among unmarried sexually active women aged 15 to 19 years who were enrolled in this study.

We found more married women (99.0%) than their unmarried counterparts (79.6%) were out of the formal educational system. In contrast, more unmarried women (52.7%) than married women (27.4%) had achieved secondary school education. These findings highlights an inverse relationship between child marriage, which is prevalent in Tanzania,[17 18] and education achievements. Child marriage is known to impact negatively on educational attainment by adolescent women and perpetuates a vicious cycle of poverty at individual, family and community levels.[1 9] However, child marriage is increasingly acknowledged as a violation of girls' human rights which must be protected by family, community and government authorities.[18]

In this study, although both married and unmarried sexually active women showed similar levels of knowledge about contraception (56.2% vs 62.4%) and self-efficacy for modern contraception use (53.0% vs 56.7%), the proportion of modern contraceptive users was far lower among married women when compared to unmarried women (19.4% vs 48.7%). Generally, in sub Saharan Africa, modern contraception use has remained low despite the rising awareness and knowledge.[5] However, the observed disparities could be pointing to presence of other factors that may have a substantial inhibitive role to contraceptive uptake particularly among married adolescent women. One of those determinants is the social pressure for adolescent women to prove their fertility immediately after marriage, [10 19] which has also been shown to be used as an anchor to win over husband's respect and stabilise marital relationship.[5 20] In this context, proving own fertility, carries

For peer review only - http://bmjopen.bmj.com/site/about/guidelines.xhtml

more weight and higher priority over higher contraceptive knowledge and self efficacy in deciding whether or not to use modern contraception.

The most commonly used contraceptive methods were implants (38.5%) among married women and male condoms (71.6%) among unmarried sexually active women. In a context of held misconceptions particularly against hormonal-based contraceptives for their perceived interference on fertility, this finding might reflect an attempt by unmarried sexually active women to preserve their fertility by opting for non-hormonal based and/or non-invasive methods. Other studies have reported that some adolescent women have chosen unsafe clandestine abortions over hormonal-based contraception.[5 21]. This finding may also reflect that unmarried sexually active adolescent women could have better access to male condoms compared to other methods. In addition, it could reflect the level of exposure and frequency of sexual intercourse, and the type of sexual relationships for unmarried adolescent women which may not require a long-term contraception method. Despite the limitation of condoms being male controlled, the double role of condoms in preventing unintended pregnancies and sexually transmitted infections including HIV, stresses the need for making condoms more accessible and advocating for their proper and regular use among unmarried sexually active adolescent women[7].

Modern contraception use among unmarried sexually active women in the study population was associated with increasing age, increasing levels of education, being in education, hearing of modern contraception from interpersonal sources and in the media in past 12 months, perceiving partner and/or friend support for contraceptive use, as well as higher knowledge about contraception and self efficacy for contraception.

We found that the odds for modern contraception use were low in the respondents who perceived that their partner and/or friends did not approve of their contraception practice compared to those

For peer review only - http://bmjopen.bmj.com/site/about/guidelines.xhtml

who perceived that their partner and/or friends did approve. Social network support has been consistently shown to influence women's decision to use contraception in various age groups and socio-cultural contexts including in sub-Saharan Africa. [5 22 23] We did not observe an association between perception of mothers support for contraceptive use and use of modern contraceptives suggesting that for unmarried adolescent women, partners and friends may be more important influencers than mothers. In addition, we found that, exposure to information about contraception from interpersonal sources or in the media in past 12 months were associated with increased odds for using modern contraception. These findings call for a need for family planning programmes to target the entire community in order to raise awareness of modern contraception and most importantly to engage male partners in support for the uptake of modern contraception.[23]

Among unmarried sexually active women, higher knowledge and self-efficacy for contraceptive use was associated with increased odds for contraceptive use. This finding, when viewed together with other significant determinants such as advancing age and being in education, underscores the spill-over effect of girls' schooling in delaying early marriage as well as its importance in giving adolescent women more time for mental and physical maturity before embarking on sexual and reproductive roles.[9] Additionally, being in education has a potential role to overcome held misconceptions against modern contraception use.[24] This agrees with another study done in rural Mwanza which found that having low education was a risk factor for unplanned pregnancy in young women. [25]

Among unmarried women, the odds for using modern contraception were found to be significantly lower among those with one or more living children when compared to those without. This being a cross sectional analysis, it could partly be telling that the unmarried women with living children are not using contraceptives in the first place hence risking early pregnancies. But it could also be telling us that unmarried women with living children are more likely to be young, out of school, with little

For peer review only - http://bmjopen.bmj.com/site/about/guidelines.xhtml

exposure to information about contraception and low self efficacy to contraception, hence low contraception use. [25 26] In addition, this finding could be pointing to the negative role of mental health issues including depression facing unmarried and/or out of school teenage mothers.[26] Despite having few studies from low and middle income countries, depression has been shown to be an independent risk factor for repeated teenage pregnancy. [27]

Strengths and limitations

In this study, we focused on adolescent women aged 15 – 19 years, a population that is often excluded or underrepresented in most of the studies on modern contraception. We also used the probability sampling approach to interview 3,511 adolescent women from 34 streets in the 15 urban and semi-urban wards of Ilemela district, Mwanza. Therefore, while it may not be possible to generalize our findings to the wider population of adolescent women aged 15 to 19 years living in urban and semi-urban wards of other regions in Tanzania, the sampling approach used allows us to generalise our findings to the wider population of adolescent women aged 15 to 19 years living in urban and semi-urban wards of Ilemela district.

However, this study has some limitations that need to be noted. The cross-sectional design makes temporal causal relationships hard to establish and reverse causality is likely, for instance, use of contraceptives may lead to higher knowledge about contraceptives.

Another limitation was the response rate of potentially eligible women which was relatively low (68.6%), mainly due to young women being at school, even after three visits in attempt to hold interviews. However, it is worth noting that the low response was not due to methodological flaws and it was more likely to involve unmarried women who were in school.

Also, the sample size of married women was small, limiting statistical power to identify determinants for contraceptive use in this group and hence we decided to drop them from the regression analysis. Lastly, we did not specifically ask the adolescent women whether they were planning on getting pregnant shortly, hence we have no data on this potential explanatory variable. In addition, pregnant women were not asked about contraception as they were not "at risk of pregnancy", the

For peer review only - http://bmjopen.bmj.com/site/about/guidelines.xhtml

same applied to those in post-partum amenorrhea. Therefore, we don't have outcome data for these two subgroups. These are fertility characteristic variables that could make a difference primarily between married and unmarried adolescent women.

CONCLUSION

In Northwest Tanzania, among married and unmarried sexually active women aged 15 to 19 years, we found strong evidence of differences in use of modern contraceptive methods according to marital status of adolescent women.

Among unmarried sexually active women, contraceptive use was significantly associated with increasing age, increasing levels of education, being in education, hearing information on modern contraception from interpersonal sources and in the media in the past 12 months, perceiving partner and/or friend support for contraception use, as well as higher knowledge and self efficacy for modern contraception.

In order to optimise their impact, sexual and reproductive health programmes aiming to increase uptake of modern contraceptives should consider the importance of being in education and social support for contraceptive use among adolescent women. Hence the need to focus intervention efforts on more vulnerable unmarried sexually active adolescent women particularly those with lower education/socioeconomic status and/or those who are already teenage mothers.

CONFLICTING INTERESTS:

The authors declare that they have no competing interests.

FUNDING:

The Bill & Melinda Gates Foundation and the Children’s Investment Fund Foundation. The funding bodies had no role in the design of the study and collection, analysis, and interpretation of data and in writing the manuscript.

For peer review only - http://bmjopen.bmj.com/site/about/guidelines.xhtml

AUTHORS' CONTRIBUTIONS:

MKN, CJA, SK, CB and AMD were involved in conception and study design. CB provided statistical expertise. MKN and CJA were involved in drafting of the manuscript. SK, CB and AMD were involved in critical revision of the manuscript for important intellectual content. All the authors were involved in final approval of the manuscript and decision to submit the manuscript for publication.

ACKNOWLEDGEMENTS:
We would like to thank all study participants for their participation and the A360 baseline study team for their dedicated work during data collection.

We also thank Itad as the lead organisation responsible for the overall A360 evaluation. Avenir Health as a partner in the overall A360 evaluation. PSI Headquarters and PSI Tanzania for their support with site selection and engagement in conversation regarding the design.

For peer review only

DATA AVAILABILITY

Individual de-identified data used for this analysis are available from AMD Aoife.Doyle@lshtm.ac.uk on reasonable request.

TABLE LEGEND

Table 1: Characteristics of married and unmarried-sexually active women aged 15-19 years in Mwanza, Tanzania[1]**.**

Table 2: Prevalence of contraceptive use among women aged 15–19 years in Mwanza, Tanzania by marital status [1].

Table 3: Factors associated with modern contraception use among unmarried sexually active women aged 15–19 years in Mwanza, Tanzania, (N=744)[£]

For peer review only - http://bmjopen.bmj.com/site/about/guidelines.xhtml

REFERENCES

1. The World Health Organisation guidelines on preventing early pregnancy and poor reproductive outcomes among adolescents in developing countries. Geneva: WHO, 2011.

2. Blum RW, Gates WH. Girlhood, not motherhood: preventing adolescent pregnnancy. New York: United Nations Populations Fund (UNFPA), 2015.

3. Family planning 2020 initiative [cited 2018 October]. Available from: https://www.familyplanning2020.org/measurement-hub#global-data accessed September 2018.

4. The UN 2030 agenda for sustainable development New York: United Nations; 2015 [cited 2019 Jan]. Available from: https://sustainabledevelopment.un.org/content/documents/21252030%20Agenda%20for%20Sustainable%20Development%20web.pdf accessed October 2018.

5. Blackstone SR, Nwaozuru U, Iwelunmor J. Factors Influencing Contraceptive Use in Sub-Saharan Africa: A Systematic Review. *Int Q Community Health Educ* 2017;37(2):79-91. doi: 10.1177/0272684X16685254

6. Nalwadda G, Mirembe F, Byamugisha J, et al. Persistent high fertility in Uganda: young people recount obstacles and enabling factors to use of contraceptives. *BMC Public Health* 2010;10:530. doi: 10.1186/1471-2458-10-530

7. Williamson LM, Parkes A, Wight D, et al. Limits to modern contraceptive use among young women in developing countries: a systematic review of qualitative research. *Reprod Health* 2009;6(3):3. doi: 10.1186/1742-4755-6-3

8. Conde-Agudelo A, Belizan JM, Lammers C. Maternal-perinatal morbidity and mortality associated with adolescent pregnancy in Latin America: Cross-sectional study. *Am J Obstet Gynecol* 2005;192(2):342-9. doi: 10.1016/j.ajog.2004.10.593

9. Wodon Q, Nayihouba A, Onagoruwa A, et al. Economic impacts of child marriage: global synthesis report. Washington DC: International Centre for Research on Women (ICRW), 2017:43-49.

For peer review only - http://bmjopen.bmj.com/site/about/guidelines.xhtml

10. Coll CVN, Ewerling F, Hellwig F, et al. Contraception in adolescence: the influence of parity and marital status on contraceptive use in 73 low-and middleincome countries *Reproductive Health* 2019(16):21-33. doi: 10.1186/s12978-019-0686-9 [published Online First: 21 Feb 2019]

11. Atchison CJ, Mulhern E, Kapiga S, et al. Evaluating the impact of an intervention to increase uptake of modern contraceptives among adolescent girls (15 - 19 years) in Nigeria, Ethiopia and Tanzania: the adolescents 360 quasi-experimental study protocol. *BMJ Open* 2018;8(5):e021834. doi: 021810.021136/bmjopen-022018-021834

12. Kavishe B, Biraro S, Baisley K, et al. High prevalence of hypertension and of risk factors for non-communicable diseases (NCDs): a population based cross-sectional survey of NCDS and HIV infection in Northwestern Tanzania and Southern Uganda. *BMC Med* 2015;13(1):126. doi: 10.1186/s12916-015-0357-9

13. Ministry of Health (MoHCDGEC) [Tanzania Mainland], Ministry of Health (MoH) [Zanzibar], National Bureau of Statistics (NBS), et al. Tanzania Demographic and Health Survey and Malaria Indicator Survey (TDHS-MIS) 2015-16. Dar es Salaam, Tanzania.: National Bureau of Statistics (NBS), 2016.

14. Central Statistical Agency (CSA) [Ethiopia], and ICF. Ethiopia Demographic and Health Survey 2016. Addis Ababa, Ethiopia and Rockville, Maryland, USA: Central Statistical Agency [Ethiopia], 2016.

15. World Health Organisation Family Planning Fact Sheet [Available from: https://www.who.int/news-room/fact-sheets/detail/family-planning-contraception accessed 09 March 2019.

16. Tanzania Equity Tool [cited 2019 16 Jan]. Available from: http://www.equitytool.org/tanzania/ accessed 18 Jan 2019.

17. Koski A, Clark S, Nandi A. Has child marriage declined in sub-Saharan Africa? An analysis of trends in 31 countries. *Popul Dev Rev* 2017;43(1):7 - 29.

For peer review only - http://bmjopen.bmj.com/site/about/guidelines.xhtml

18. (Tanzania) MoH. National survey on the drivers and consequences of child marrige in Tanzania. Dar es Salaam - Tanzania: Ministry of Health (MoHCDGEC), 2017.

19. UNDESA. Trends in the contraceptive use worldwide. New York: United Nations Department of Economic and Social Affairs (UNDESA - Population Division). 2015.

20. Eaton L, Flisher AJ, Aar LE. Unsafe sexual behaviour in south African youth. *Soc Sci Med* 2002;56(2003):149 - 65.

21. Plummer ML, Wamoyi J, Nyalali K, et al. Aborting and suspending pregnancy in rural Tanzania: an ethnography of young people's beliefs and practices. *Stud Fam Plann* 2008;39(4):281-92.

22. Omideyi AK, Akinyemi AI, Aina OI, et al. Contraceptive practice, unwanted pregnancies and induced abortion in southwest Nigeria. *Global public health* 2011;6 Suppl 1:S52-72. doi: 10.1080/17441692.2011.594073

23. Samandari G, Speizer IS, O'Connell K. The role of social support and parity on contraceptive use in Cambodia. *Int Perspect Sex Reprod Health* 2010;36(3):122-31.

24. Mardi A, Ebadi A, Shahbazi S, et al. Factors influencing the use of contraceptives through the lens of teenage women: a qualitative study in Iran. *BMC Public Health* 2018;18(1):202. doi: 10.1186/s12889-018-5116-3

25. Calvert C, Baisley K, Doyle AM, et al. Risk factors for unplanned pregnancy among young women in Tanzania. *J Fam Plann Reprod Health Care* 2013;39(4):e2. doi: 10.1136/jfprhc-2012-100389

26. SmithBattle L, Freed P. Teen Mothers' Mental Health. *MCN The American journal of maternal child nursing* 2016;41(1):31-6; quiz E3-4. doi: 10.1097/NMC.0000000000000198

27. Maravilla JC, Betts KS, e Cruz C, et al. Factors influencing repeated teenage pregnancy: A review and meta analysis. *Am J Obstet Gynecol* 2017;19e

For peer review only - http://bmjopen.bmj.com/site/about/guidelines.xhtml

Reporting checklist for cross sectional study.

Based on the STROBE cross sectional guidelines.

Instructions to authors

Complete this checklist by entering the page numbers from your manuscript where readers will find each of the items listed below.

Your article may not currently address all the items on the checklist. Please modify your text to include the missing information. If you are certain that an item does not apply, please write "n/a" and provide a short explanation.

Upload your completed checklist as an extra file when you submit to a journal.

In your methods section, say that you used the STROBE cross sectional reporting guidelines, and cite them as:

von Elm E, Altman DG, Egger M, Pocock SJ, Gotzsche PC, Vandenbroucke JP. The Strengthening the Reporting of Observational Studies in Epidemiology (STROBE) Statement: guidelines for reporting observational studies.

		Reporting Item	Page Number
Title	#1a	Indicate the study's design with a commonly used term in the title or the abstract	1 & 2
Abstract	#1b	Provide in the abstract an informative and balanced summary of what was done and what was found	2

For peer review only

Background / rationale	#2	Explain the scientific background and rationale for the investigation being reported	4 & 5
Objectives	#3	State specific objectives, including any prespecified hypotheses	5
Study design	#4	Present key elements of study design early in the paper	5
Setting	#5	Describe the setting, locations, and relevant dates, including periods of recruitment, exposure, follow-up, and data collection	5
Eligibility criteria	#6a	Give the eligibility criteria, and the sources and methods of selection of participants.	6
	#7	Clearly define all outcomes, exposures, predictors, potential confounders, and effect modifiers. Give diagnostic criteria, if applicable	8 - 10
Data sources / measurement	#8	For each variable of interest give sources of data and details of methods of assessment (measurement). Describe comparability of assessment methods if there is more than one group. Give information separately for exposed and unexposed groups if applicable.	8 - 9
Bias	#9	Describe any efforts to address potential sources of bias	6 & 7
Study size	#10	Explain how the study size was arrived at	7
Quantitative variables	#11	Explain how quantitative variables were handled in the analyses. If applicable, describe which groupings were chosen, and why	8 - 10

For peer review only

Statistical methods	#12a	Describe all statistical methods, including those used to control for confounding	9
	#12b	Describe any methods used to examine subgroups and interactions	
	#12c	Explain how missing data were addressed	
	#12d	If applicable, describe analytical methods taking account of sampling strategy	9
	#12e	Describe any sensitivity analyses	
Participants	#13a	Report numbers of individuals at each stage of study—eg numbers potentially eligible, examined for eligibility, confirmed eligible, included in the study, completing follow-up, and analysed. Give information separately for for exposed and unexposed groups if applicable.	11
	#13b	Give reasons for non-participation at each stage	11
	#13c	Consider use of a flow diagram	n/a
Descriptive data	#14a	Give characteristics of study participants (eg demographic, clinical, social) and information on exposures and potential confounders. Give information separately for exposed and unexposed groups if applicable.	11
	#14b	Indicate number of participants with missing data for each variable of interest	11,12,13
Outcome data	#15	Report numbers of outcome events or summary measures.	14

For peer review only

		Give information separately for exposed and unexposed groups if applicable.	
Main results	#16a	Give unadjusted estimates and, if applicable, confounder-adjusted estimates and their precision (eg, 95% confidence interval). Make clear which confounders were adjusted for and why they were included	17- 19
	#16b	Report category boundaries when continuous variables were categorized	11 , 12
	#16c	If relevant, consider translating estimates of relative risk into absolute risk for a meaningful time period	n/a
Other analyses	#17	Report other analyses done—e.g., analyses of subgroups and interactions, and sensitivity analyses	n/a
Key results	#18	Summarise key results with reference to study objectives	23
Limitations	#19	Discuss limitations of the study, taking into account sources of potential bias or imprecision. Discuss both direction and magnitude of any potential bias.	3 & 24
Interpretation	#20	Give a cautious overall interpretation considering objectives, limitations, multiplicity of analyses, results from similar studies, and other relevant evidence.	25
Generalisability	#21	Discuss the generalisability (external validity) of the study results	3 & 24
Funding	#22	Give the source of funding and the role of the funders for the present study and, if applicable, for the original study on which	25

For peer review only

the present article is based

The STROBE checklist is distributed under the terms of the Creative Commons Attribution License CC-BY. This checklist can be completed online using https://www.goodreports.org/, a tool made by the EQUATOR Network in collaboration with Penelope.ai

For peer review only

BMJ Open

Modern contraceptive use among sexually active women aged 15 to 19 years in north-western Tanzania: results from the Adolescent 360 (A360) baseline survey

Journal:	*BMJ Open*
Manuscript ID	bmjopen-2019-030485.R2
Article Type:	Research
Date Submitted by the Author:	28-Jun-2019
Complete List of Authors:	Nsanya, Mussa; Mwanza Intervention Trials Unit, Atchison, Christina; London School of Hygiene and Tropical Medicine Faculty of Epidemiology and Population Health, MRC Tropical Epidemiology Group Bottomley, Christian ; London School of Hygiene and Tropical Medicine Faculty of Epidemiology and Population Health, MRC Tropical Epidemiology Group Doyle, Aoife; London School of Hygiene and Tropical Medicine Faculty of Epidemiology and Population Health, MRC Tropical Epidemiology Group Kapiga, Saidi ; London School of Hygiene and Tropical Medicine, Infectious Disease Epidemiology; London School of Hygiene and Tropical Medicine Faculty of Epidemiology and Population Health, MRC Tropical Epidemiology Group
**Primary Subject Heading**:	Public health
Secondary Subject Heading:	Reproductive medicine
Keywords:	Adolescents, Contraception, Family Planning, Reproductive health, Africa

SCHOLARONE™
Manuscripts

TITLE

Modern contraceptive use among sexually active women aged 15 to 19 years in north-western Tanzania: results from the Adolescent 360 (A360) baseline survey

Key words: Adolescents, Contraception, Family Planning, Reproductive health, Africa

Authors:

Mussa Kelvin Nsanya[1*], Christina J. Atchison[2], Christian Bottomley[2], Aoife M. Doyle[2], and Saidi H. Kapiga[1,3]

Institutional Affiliations:

1.Mwanza Intervention Trials Unit/National Institute for Medical Research, P.O. Box 11936, Mwanza, Tanzania

2. MRC Tropical Epidemiology Group, London School of Hygiene and Tropical Medicine, Keppel Street, London WC1E 7H, UK

3. Department of Infectious Disease Epidemiology, London School of Hygiene and Tropical Medicine, Keppel Street, London WC1E 7H, UK

*Corresponding author: mussa.nsanya@mitu.or.tz , kelvin.nsanya@gmail.com

For peer review only

ABSTRACT

Objectives: To describe differences in modern contraceptive use among adolescent women aged 15 to 19 years according to their marital status and to determine factors associated with modern contraceptive use among sexually active women in this population.

Design: Cross-sectional analysis of Adolescent 360 (A360) evaluation baseline survey.

Setting: The 15 urban and semi-urban wards of Ilemela district, Mwanza region, North-western Tanzania.

Participants: Adolescent women aged 15 – 19 years who were living in the study site from August 2017 to February 2018 and who provided informed consent. Women were classified as married if they had a husband or were living as married. Unmarried women were classified as sexually active if they reported having sexual intercourse in the past 12 months.

Outcome measure: Prevalence of modern contraceptive (mCPR) among adolescent women aged 15 – 19 years.

Results: Data was available for 3,511 women aged 15-19 years, of which 201(5.7%) were married and 744 (22.5%) were unmarried-sexually active. We found strong evidence of differences in use of modern contraceptive methods according to marital status of adolescent women. Determinants of modern contraception use among unmarried-sexually active women were increasing age, increasing level of education, being in education, hearing of modern contraception from interpersonal sources or in the media in the past 12 months, perceiving partner and/or friends support for contraceptive use, as well as higher knowledge and self efficacy for contraception.

Conclusions: Sexual and reproductive health programmes aiming to increase uptake of modern contraceptives in this population of adolescent women should consider the importance of girl's education and social support for contraceptive use particularly among unmarried sexually active women.

For peer review only - http://bmjopen.bmj.com/site/about/guidelines.xhtml

For peer review only

Strengths and limitations of this study

- We focused on adolescent women aged 15 – 19 years, a population that is often excluded or underrepresented in most of the studies on modern contraception.
- We used the probability sampling approach to interview 3,511 adolescent women from 34 streets in the 15 urban and semi-urban wards of Ilemela district, Mwanza. Therefore, while it may not be possible to generalize our findings to the wider population of adolescent women aged 15 to 19 years living in similar urban and semi-urban wards of other regions in Tanzania, the sampling approach used allows us to generalise our findings to the wider population of adolescent women aged 15 to 19 years living in urban and semi-urban wards of Ilemela district.
- The main limitations are: the cross-sectional design makes temporal causal relationships hard to establish and reverse causality is likely, for example, use of contraceptives may lead to higher knowledge about contraceptives.
- Also, because of the small sample size of married women, the study had limited power to identify determinants for contraceptive use in this group and hence the decision to drop them from the regression analysis.
- We did not specifically ask the adolescent women whether they were planning on getting pregnant shortly, hence we have no data on this potential explanatory variable. In addition, pregnant women were not asked about contraception as they were not "at risk of pregnancy", the same applied to those in post-partum amenorrhea. So we don't have outcome data for these two subgroups. These are fertility characteristic variables that could make a difference primarily between married and unmarried adolescent girls.

For peer review only - http://bmjopen.bmj.com/site/about/guidelines.xhtml

INTRODUCTION

Globally, approximately 16 million women aged 15 to 19 years give birth each year and 95% of these births take place in low and middle income countries.[1 2] The global community, through the Family Planning 2020 (FP2020) initiative, is committed to increase new contraceptive users to 120 million in 69 developing countries (including Tanzania) by 2020. [3] This initiative would also support the objectives of the United Nation's Sustainable Development Goal (SDG) 3 on health and wellbeing for all and SDG 5 on gender equality which also embodies sexual and reproductive health at the heart of global efforts to sustainable development particularly in low and middle income countries.[4]

Modern contraceptive use remains unacceptably low in sub-Saharan Africa despite increasing awareness and knowledge about contraception.[5 6] For instance, 69% and 59% of the women aged 15 to 19 years in United Kingdom and United States of America respectively report using a modern contraceptive at the last time they had sexual intercourse compared to 12% in Mali and 21% in Tanzania.[7] The low uptake of modern contraceptives particularly among women aged 15 to 19 years contributes significantly to high rates of adolescent pregnancies and poor health outcomes including maternal morbidity and mortality, and neonatal and under-five child mortality.[1 5 8] In addition, there are other severe social and economic consequences to adolescent women, their families and whole society including not reaching their potential for educational achievement, and not getting a paid job which usually leads into a vicious cycle of poverty. [1 9]

Most studies to date have focused on the factors that prevent women of reproductive age (15 – 49 years) from using modern contraceptives.[5] In such studies adolescent women are usually underrepresented despite facing disproportionate medical, social and economic impact of unintended pregnancies.[1] In order for the goals of FP2020 and SDG 3 and 5 to be achieved, more information is required from studies which examine factors associated with contraceptive use

For peer review only - http://bmjopen.bmj.com/site/about/guidelines.xhtml

among adolescent women particulary in developing countries, including Tanzania. Moreover, demand for and access to modern contraceptives among adolescent women aged 15 to 19 years are known to differ with the women's marital status. [10] It is therefore important to describe differences according to marital status to optimise access and use of modern contraception.

Adolescents 360 (A360) is an initiative being rolled out across Ethiopia, Nigeria and Tanzania, aiming to increase uptake of voluntary modern contraception among sexually active women aged 15 to 19 years.[11] Using baseline survey data collected as part of A360 programme evaluation, we describe differences in modern contraceptive use among adolescent women aged 15 to 19 years according to their marital status; and determine factors associated with modern contraceptive use among unmarried sexually active women in this population in Mwanza, Tanzania.

METHODS

Study design and settings

Between August 2017 and February 2018, we conducted a cross-sectional baseline survey among women aged 15 to 19 years in Mwanza city, Tanzania. The survey was part of a comprehensive outcome evaluation to assess the impact of the A360 programme on a number of sexual and reproductive health outcomes, primarily uptake of voluntary modern contraception among sexually active women aged 15 to 19 years.[11]

In Tanzania, A360 is being implemented in 16 administrative regions. This survey was conducted in fifteen urban and semi-urban wards of Ilemela district, Mwanza region.[11] Ilemela district covers the northern part of Mwanza city and is comprised of 19 wards, of which 4 are rural wards, 6 are semi-urban and 9 are urban wards. Each ward is administratively divided into a number of neighbourhoods, called 'streets'.

For peer review only - http://bmjopen.bmj.com/site/about/guidelines.xhtml

This survey (which is part of the intervention evaluation) was set in urban and semi-urban wards of Ilemela district because Population Services International (PSI), the A360 intervention implementers in Tanzania, are focusing their efforts in more densely populated areas.

Study population

Women were included in the study if they were 15 to 19 years old; living in the study sites at the time of the survey; and voluntarily provided informed consent. Women were classified as married if they reported that they had a husband or were living as married with a cohabiting male partner.

Informed consent

Written informed consent was obtained from all participants. A parental consent waiver was granted for unmarried women aged 15 to 17 years, given the sensitive nature of the topics discussed. Married women under 18 years of age were considered emancipated and did not require parental consent in addition to their own voluntary consent.

Ethics approval

The study was approved by the Tanzania National Health Research Ethics review sub-committee of the National Institute for Medical Research (Ref: NIMR/HQ/R8a/Vol.IX/2549), and the research ethics committee of the London School of Hygiene & Tropical Medicine (LSHTM, Ref: 14145).

Sampling strategy and sample size

A cluster sampling design was used. The primary sampling unit (PSU) for the survey was a 'street', the smallest administrative unit similar to a neighbourhood or a localised and delineated group of people. All 15 urban or semi-urban wards of Ilemela district were included in the survey. Each ward has an estimated eight to ten streets. A simple random sample of 34 'streets' was selected across the 15 urban and semi-urban wards of Ilemela district. As per study protocol, in the first eight

For peer review only - http://bmjopen.bmj.com/site/about/guidelines.xhtml

For peer review only

'streets', we randomly selected 50 GPS coordinates using ArcGIS software version 9.3 (Esri, Redlands, USA). All households whose front door was located within a radius of 20 meters around the GPS coordinates were visited and all eligible consenting women aged 15 to 19 years residing in these households were invited to be interviewed. This approach was considered due to lack of detailed lists of households in the streets which could serve as a sampling frame. [12] As fewer eligible women than predicted were surveyed in each cluster using this sampling strategy, in the remaining 26 'streets' we visited all households and administered the questionnaire to all eligible and consenting women aged 15 to 19 years. Our target sample size for the baseline survey was 3,314 women aged 15 to 19 years. Sample size and power calculations were conducted ahead of deciding on the number of streets to sample. The estimated sample size had 90% power to detect a 6% increase in prevalence of modern contraceptive use in presence of A360 intervention for 24 months. [11]

If potentially eligible participants were not available at the first visit, two further revisits were made to attempt to hold interviews.

Participants and Public Involvement

We sought permission from local government authorities in the wards where the study took place as well as from individual participants prior to enrollment. Additionally, we have communicated the A360 baseline survey report to local government officials in Ilemela district, Mwanza

Tool for baseline survey

The questionnaire was adapted from various research instruments that have been used in the target countries including the Tanzania Demographic and Health Survey (DHS)[13] and FP2020 survey instruments[3]. Questionnaires were administered face-to-face by female interviewers aged between 18 and 26 years using pre-programmed tablet computers.[11]

For peer review only - http://bmjopen.bmj.com/site/about/guidelines.xhtml

The questionnaire had three components: (1) socio-demographic characteristics, (2) fertility characteristics and preferences, and (3) contraceptive knowledge, attitudes and practices. Only respondents who reported sexual intercourse in the last 12 months were considered sexually active hence asked questions about contraceptive use.[11]

Study outcome

The prevalence of modern contraceptives (mCPR) among married women aged 15 to 19 years was defined as per the DHS definition[14]:

$$\frac{\text{Number of married 15–19-year-old women reporting use of modern contraceptives at the time of the survey}}{\text{Number of married 15–19-year-old women}}$$

mCPR among unmarried-sexually active women aged 15 to 19 years was defined as follows:

$$\frac{\text{Number of unmarried-sexually active* women aged 15–19-year-old reporting use of modern contraceptives at the time of the survey}}{\text{Number of unmarried-sexually active* unmarried women aged 15–19-year-old}}$$

*self-reported that they were sexually active in the 12 months prior to the survey

Modern contraception was defined to include the following: male and female sterilisation, contraceptive implants, intrauterine contraceptive devices (IUCD), injectables, oral contraceptive

For peer review only - http://bmjopen.bmj.com/site/about/guidelines.xhtml

pill, emergency contraceptive pill, male condom, female condom, Standard Days Method (SDM), Lactational Amenorrhoea Method (LAM), diaphragm, spermicides, foams and jelly.[15]

Statistical analysis

Data analysis was conducted in Stata 15. We used sampling weights and robust standard errors to account for the clustered sampling design.

Descriptive data analysis was done for both married and unmarried women. Logistic regression was performed for unmarried-sexually active women only due to small sample size for married women. We obtained odds ratios (OR) for the association of each explanatory variable with use of modern contraception. Wald tests adjusted for the clustered sampling design were used at each step of the analysis. The associations between mCPR and age and between mCPR and religion were not adjusted for other explanatory variables. Age and religion were considered a priori potential confounders for the associations between mCPR and highest education level achieved, currently being in education, type of area of residence (urban or semi-urban) and socioeconomic position. The remaining explanatory variables with p-value < 0.2 in the univariate analysis, were investigated one-by-one in multivariate regression models adjusted for age, religion, highest education level achieved, currently being in education and socioeconomic position. Variables with p value < 0.05 in the adjusted analysis were considered to be associated with mCPR. This strategy allowed us to assess the effect of variables adjusted for distal a priori potential confounders.

Socioeconomic position was created from a series of questions about household items, dwelling materials and access to a bank account. The variable was generated using the “Tanzania Equity Tool” which uses different weights attached to each answer to create a composite score which was then split into quintiles according to the national thresholds.[16]

For peer review only - http://bmjopen.bmj.com/site/about/guidelines.xhtml

Knowledge about contraception was assessed through the respondents affirmative report to the following five questions: (1) preventing unintended pregnancies is a benefit of contraception, (2) preventing abortions is a benefit of contraception, (3) some contraceptive methods reduce sexually transmitted infections/HIV, (4) modern contraception can help with child spacing, and (5) using modern contraception can allow a woman to complete her education, take up better economic opportunities and fulfil her potential.

Holding misconceptions was assessed by asking respondents whether they agreed with the following four statements: (1) use of a long-acting reversible contraceptive can make adolescent women permanently infertile, (2) changes to normal menstrual bleeding patterns, which is caused by some contraceptives, are harmful to health, (3) modern contraceptives can make adolescent women permanently fat, and (4) adolescent women who use family planning/birth spacing may become promiscuous.

Self-efficacy for contraception was assessed through four questions relating to the woman's ability to access and use contraception: (1) felt able to start a conversation with her partner about contraception, (2) felt able to use a method of contraception even if her partner did not want her to, (3) felt able to obtain information on contraception services and products if she needed to, and (4) felt able to obtain a contraceptive method if she decided to use one.

Variables for contraception knowledge, holding misconceptions, and self-efficacy were created as scores from 0 - 5 for knowledge, and 0 - 4 for holding misconceptions and self-efficacy based on the overall score for each individual statement in each category. A score of 1 was given if the respondent agreed with the statement and 0 if she disagreed or answered "*don't know*". A maximum score of 5 for knowledge and 4 for self-efficacy would indicate that the respondent correctly agreed with all five knowledge statements and felt able to achieve all four self-efficacy behaviours. A maximum

For peer review only - http://bmjopen.bmj.com/site/about/guidelines.xhtml

score of 4 for holding misconceptions would be interpreted as believing all four myth statements about contraception.

RESULTS

A total of 14,138 households were identified and 99.6% were interviewed to obtain information on household members. A total of 5,121 potentially eligible women aged 15–19 years were identified from 3,963 households (28.1% of all interviewed households); 68.6% (3,511) of potentially eligible women were interviewed, of whom 5.7% (201/3,511) were married. Overall, 22.5% (744/3,310) of unmarried women had been sexually active during the 12 months preceding the survey. The most common reason for a potentially eligible woman not interviewed was being absent or unavailable at home after a maximum of three visits mainly due to attending school.

In Table 1, we present the characteristic of married and unmarried-sexually active women aged 15 to 19 years in the study population. The median age was 19 years for married women and 18 years for unmarried-sexually active women. The majority of respondents were Christian (married: 79.7%, unmarried: 84.5%) and were not currently pursuing educational training (married: 99.0%, unmarried: 79.6%). The highest level of education achieved by majority of married women was primary education (65.2%) while the majority of unmarried-sexually active women had achieved secondary level education (52.7%). Most respondents had moderate knowledge about contraception (married: 56.2%, unmarried: 62.4%) and a moderate self-efficacy for contraception (married: 53.0%, unmarried: 56.7%).

In Table 2, we present the prevalence of contraceptive use among women aged 15 to 19 years in Mwanza by their marital status. Overall, 19.4% of married respondents and 48.7% of unmarried-sexually active respondents were using a modern method of contraception. Of those reporting using a modern method of contraception, implants (38.5%) were the most widely used method by married

For peer review only - http://bmjopen.bmj.com/site/about/guidelines.xhtml

women, followed by injectables (23.1%) and Standard Days Method (15.4%). Male condoms (71.6%) were the most widely used modern contraceptive method by unmarried-sexually active women, followed by Standard Days Method (15.8%). Overall, there was strong evidence of differences in use of modern contraceptive methods according to marital status of adolescent women (Table 2).

For peer review only

Table 1. Characteristics of married and unmarried-sexually active women aged 15-19 years in Mwanza, Tanzania[1].

Characteristic	Married, N=201	Unmarried, N=744	p value
**Sociodemographic factors**	N (%)	N (%)	
**Age (years)**			
15	2 (1.0)	62 (8.3)	
16	4 (2.0)	82 (11.0)	
17	24 (11.9)	161 (21.6)	
18	69 (34.3)	199 (26.8)	
19	102 (50.8)	240 (32.3)	<0.0001
**Age (years)[¥]**	19 (18,19)	18 (17-19)	<0.0001
**Religion**			
Catholic	61 (30.4)	309(41.5)	
Protestant / Other Christian	99 (49.3)	320 (43.0)	
Muslim	38 (18.9)	112 (15.1)	
No religion	3 (1.5)	3 (0.40)	0.04
**Highest level of education achieved**			
No education	15 (7.5)	21 (2.8)	
Primary education	131 (65.2)	320 (43.0)	
Secondary education	55 (27.4)	392 (52.7)	
University education	0	11 (1.5)	<0.0001
**Currently in educational training programme**			
Yes	2 (1.0)	152 (20.4)	
No	199 (99.0)	592 (79.6)	<0.0001
**Type of area of residence**			
Semi-urban	85 (42.3)	290 (39.0)	
Urban	116 (57.7)	454 (61.0)	0.43
**Socio-economic level**			
Lowest Quintile	38 (22.1)	87 (15.5)	
2nd Lowest Quintile	55 (32.0)	131 (23.4)	
Middle Quintile	31 (18.0)	82 (14.6)	
2nd Highest Quintile	36 (20.9)	125 (22.3)	
Highest Quintile	12 (7.0)	135 (24.1)	0.0002
**Exposure to information about contraception**			
**Heard about contraception in the media in last 12 months**	59 (29.4)		
Yes	142 (70.7)	309 (41.5)	

For peer review only - http://bmjopen.bmj.com/site/about/guidelines.xhtml

No		435 (58.5)	<0.0001
**Heard about contraception from health sector sources in last 12 months**			
Yes	122 (60.7)	213 (28.6)	
No	79 (39.3)	531 (71.4)	<0.0001
**Heard about contraception from interpersonal sources in last 12 months**			
Yes	100 (49.8)	487 (65.5)	
No	101 (50.3)	257 (34.5)	0.0001
**Knows a place where or person from whom she would feel comfortable accessing contraception**			
Yes	113 (61.1)	400 (53.8)	
No	72 (38.9)	343 (46.2)	0.14
**Social networks**			
**Perceives that partner supports her using contraception**			
Yes	116 (62.7)	430 (60.2)	
No	45 (24.3)	140 (19.6)	
Don't know	24 (13.0)	144 (20.2)	0.04
**Perceives that mother supports her using contraception**			
Yes	89 (50.9)	299 (42.4)	
No	53 (30.3)	190 (26.9)	
Don't know	33 (18.9)	217 (30.7)	0.03
**Perceives that friends supports her using contraception**			
Yes	85 (46.2)	430 (58.3)	
No	38 (20.7)	100 (13.6)	
Don't know	61 (33.2)	207 (28.1)	0.02
**Individual knowledge, attitudes and behaviours**			
**Knowledge about contraception score***			
0-1	21 (10.5)	37 (5.0)	
2-3	67 (33.3)	243 (32.7)	
4-5	113 (56.2)	464 (62.4)	0.02
**Misconceptions about contraception score****			
0-1	83 (41.3)	258 (34.7)	
2-3	75 (37.3)	375 (50.4)	
4	43 (21.4)	111 (14.9)	0.04
**Self-efficacy for contraception score*****			
0-1	15 (8.1)	57 (7.7)	
2-3	72 (38.9)	265 (35.7)	
4	98 (53.0)	421 (56.7)	0.67
**Timing of most recent sexual activity**			
Within past week	86 (42.8)	48 (6.5)	
Within past month	52 (25.9)	207 (27.8)	
Within past year	63 (31.3)	489 (65.7)	<0.0001
**Number of living children**			
No children	97 (48.3)	638 (85.8)	
1 child or more	104 (51.7)	106 (14.3)	<0.0001

[1] The figures refers to N (%). Numbers and percentages may not match exactly because the analysis used sampling weights to account for the sampling design.

[¥] Median (Inter quartile range)

***** Scored based on the responses to the following five questions: (1) preventing unintended pregnancies is a benefit of contraception, (2) preventing abortions is a benefit of contraception, (3) some contraceptive methods reduce sexually

For peer review only

transmitted infections/HIV, (4) modern contraception can help with child spacing, and (5) using modern contraception can allow a woman to complete her education, take up better economic opportunities and fulfil her potential.

** Scored based on the responses to the following four questions: (1) use of a long-acting reversible contraceptive can make adolescent women permanently infertile, (2) changes to normal menstrual bleeding patterns, which is caused by some contraceptives, are harmful to health, (3) modern contraceptives can make adolescent women permanently fat, and (4) adolescent women who use family planning/birth spacing may become promiscuous.

*** Scored based on the responses to the following four questions: (1) felt able to start a conversation with her partner about contraception, (2) felt able to use a method of contraception even if her partner did not want her to, (3) felt able to obtain information on contraception services and products if she needed to, and (4) felt able to obtain a contraceptive method if she decided to use one.

Table 2. Prevalence of contraceptive use among women aged 15–19 years in Mwanza, Tanzania by marital status [1].

Characteristic	Married, N=201[2]	Unmarried, N=744[2]	p value
**Any method**	20.4 (13.9-28.9)	50.7 (47.7-53.6)	<0.0001
**Any modern method[3]**	19.4 (13.4-27.3)	48.7 (45.8-51.5)	
**Any traditional method**	1.0 (0.22-4.4)	2.0 (1.3-3.0)	
**Not currently using**	79.6 (71.2-86.1)	49.3 (46.4-52.3)	<0.0001
Total	100.0	100.0	
**Modern method**			
Implant	38.5 (21.4-58.9)	4.4 (2.4-7.9)	
IUCD	7.7 (2.5-21.5)	0.28 (0.03-2.3)	
Injectables	23.1 (10.8-42.7)	5.8 (3.8-8.7)	
Oral contraceptive pill	2.6 (0.31-18.0)	0.55 (0.13-2.4)	
Emergency pill	0	0.55 (0.12-2.4)	
Male condom	7.7 (2.4-22.2)	71.6 (66.9-75.8)	
Standard Days Method	15.4 (5.2-37.6)	15.8 (12.1-20.3)	
Other modern method	5.1 (1.4-16.6)	1.1 (0.37-3.2)	<0.0001
Total	100.0	100.0	

1 Figures are % (95% confidence intervals) taking into account sampling weights to account for the sampling design.
2 Unmarried girls who report sexually activity in last 12 months; all married girls in Ilemela district.
3 Modern methods include female sterilisation, male sterilisation, contraceptive pill (oral contraceptives), IUCD, injectables (Depo-Provera), implants (Norplant), female condom, male condom, diaphragm, contraceptive foam and contraceptive jelly, LAM, SDM, cycle beads.

In Table 3, we present factors associated with use of modern contraceptive methods among unmarried-sexually active women aged 15 – 19 years in Mwanza, Tanzania.

Socio-demographic factors

Age, religion, level of education, being in an educational programme and socio-economic position were all associated with use of modern contraceptive methods in univariate analysis (p-value <0.2).

For peer review only - http://bmjopen.bmj.com/site/about/guidelines.xhtml

The odds of using modern contraception increased with age (adjusted OR (adjOR) 1.2, 95% CI, 1.1-1.4). Following adjustment for age and religion, there was strong evidence that unmarried-sexually active women who had reached university level education were three times more likely to use modern contraception compared to those with no education (adjOR 3.0, 95% CI,1.0, 9.0 , p-value 0.004) and those who were not in education had significantly lower odds of using modern contraception compared to those in education (adjOR 0.52, 95% CI 0.36-0.75).

Exposure to information about contraception

Hearing about contraception in the media in the last 12 months or from an interpersonal source, and knowing of a place or person from whom respondent would feel comfortable accessing contraception were significantly associated with using modern contraception in univariate analysis (p-value < 0.2).

After adjusting for socio-demographic variables, the odds of using modern contraception were significantly lower for unmarried-sexually active women who had not heard about contraception in the media in the last 12 months (adjOR 0.58, 95% CI 0.35-0.95) or from interpersonal sources (adjOR 0.61, 95% CI 0.42-0.90) compared to those that had heard this information.

Social network factors

Perceived support from partner, mother and friends for using contraception were associated with using modern contraception in univariate analysis (p-value <0.2). After adjusting for socio-demographic variables, the odds of using modern contraception were lower for unmarried-sexually active women who perceived that their partners did not support their use of contraception (adjOR 0.29, 95% CI 0.21-0.42) and similarly for those who perceived lack of support from their friends (adjOR 0.55, 95% CI 0.34-0.88) compared to those who perceived their support.

For peer review only

Individual knowledge, attitudes and behaviour factors

Knowledge about contraception, holding misconceptions against contraception, self-efficacy for contraception and number of living children were associated with using modern contraception in univariate analysis (p-value <0.2). After adjusting for demographic variables, the odds of using modern contraception were significantly lower in unmarried-sexually active women with one or more living children compared to those without a child (adjOR 0.57, 95%CI 0.39-0.85). The odds of using modern contraception increased with higher knowledge about contraception (adjOR 2.4, 95% CI 1.2-4.6) and higher self-efficacy for using contraception (adjOR 2.4, 95% CI 1.5-4.1).

For peer review only

For peer review only - http://bmjopen.bmj.com/site/about/guidelines.xhtml

Table 3. Factors associated with modern contraception use among unmarried sexually active women aged 15–19 years in Mwanza, Tanzania, (N=744)£

Exposure category	No.	Prevalence, n (%)	Unadjusted OR (95% CI)	p value	adjusted OR (95% CI)	p value
**Sociodemographic factors**						
**Age (years)**						
15	62	20 (32.3)				
16	82	30 (36.6)				
17	161	79 (49.1)				
18	199	107 (53.8)				
19	240	126 (52.5)				
Per Year Increase			1.2 (1.1-1.4)	0.01		
**Religion**						
Catholic	309	145 (46.9)	1			
Protestant/Other Christian	320	149 (46.6)	0.99 (0.77-1.3)			
Muslim	112	66 (58.9)	1.6 (1.1-2.4)			
No religion	3	2 (66.7)	2.3 (0.50-10.2)	0.10		
**Highest educational achieved[1]**						
No education	21	7 (33.3)	1		1	
Primary	320	127 (39.7)	1.3 (0.39-4.5)		1.4 (0.40-4.7)	
Secondary	392	221 (56.4)	2.6 (0.83-8.0)		2.5 (0.78-8.1)	
University	11	7 (63.6)	3.5 (1.2-9.8)	0.0006	3.0 (1.0-9.0)	0.004
**Currently in educational training[1]**						
Yes	152	89 (58.6)	1		1	
No	592	273 (46.1)	0.61 (0.43-0.86)	0.008	0.52 (0.36-0.75)	0.002
**Type of area of residence**						
Semi-urban	290	140 (48.3)	1			
Urban	454	222 (48.9)	1.0 (0.82-1.3)	0.82		
**Socio-economic level[1]**						
Lowest Quintile	87	36 (41.4)	1		1	
2nd Lowest Quintile	131	62 (47.3)	1.3 (0.71-2.3)		1.2 (0.70-2.1)	
Middle Quintile	82	43 (52.4)	1.6 (0.80-3.0)		1.5 (0.72-3.1)	
2nd Highest Quintile	125	59 (47.2)	1.3 (0.70-2.3)		1.2 (0.60-2.3)	
Highest Quintile	135	79 (58.5)	2.0 (1.1-3.6)	0.08	1.9 (1.1-3.4)	0.09
**Exposure to information about**						

For peer review only - http://bmjopen.bmj.com/site/about/guidelines.xhtml

**contraception**						
**Heard about contraception in the media in last 12months[2]**						
Yes	309	174 (56.3)	1		1	
No	435	188 (43.2)	0.59 (0.42-0.83)	0.004	0.58 (0.35-0.95)	0.03
**Heard about contraception from health sector sources in last 12months**						
Yes	213	101 (47.4)	1			
No	531	261 (49.2)	1.1 (0.71-1.6)	0.73		
**Heard about contraception from interpersonal sources in last 12months[2]**						
Yes	487	261 (53.6)	1		1	
No	257	101 (39.3)	0.56 (0.40-0.78)	0.002	0.61 (0.42-0.90)	0.01
**Know of a place where or person from whom she would feel comfortable accessing contraception[2]**						
Yes	400	213 (53.3)	1		1	
No	343	149 (43.4)	0.67 (0.50-0.92)	0.01	0.69 (0.46-1.0)	0.07
**Social networks**						
**Perceives that partner supports her using contraception[2]**						
Yes	430	264 (61.4)	1		1	
No	140	40 (28.6)	0.25 (0.18-0.35)		0.29 (0.21-0.42)	
Don't know	144	53 (36.8)	0.37 (0.24-0.55)	<0.0001	0.32 (0.20-0.52)	<0.0001
**Perceives that mother supports her using contraception[2]**						
Yes	299	160 (53.5)	1		1	
No	190	89 (46.8)	0.77 (0.53-1.1)		0.87 (0.56-1.4)	
Don't know	217	97 (44.7)	0.70 (0.54-0.92)	0.05	0.73 (0.48-1.1)	0.32
**Perceives that friends supports her using contraception[2]**	430	240 (55.8)				
Yes	100	44 (44.0)	1		1	
No	207	76 (36.7)	0.62 (0.45-0.86)		0.55 (0.34-0.88)	

For peer review only - http://bmjopen.bmj.com/site/about/guidelines.xhtml

Don't know			0.46 (0.33-0.63)	0.0004	0.42 (0.29-0.61)	0.0004
**Individual knowledge, attitudes and behaviours**						
**Knowledge about contraception[2,*]**			1		1	
0-1	37	12 (32.4)	1.6 (0.98-2.5)		1.9 (1.0-3.4)	
2-3	243	104 (42.8)	2.4 (1.4-4.0)	0.01	2.4 (1.2-4.6)	0.05
4-5	464	246 (53.0)				
**Misconceptions about contraception[2,**]**						
0-1	258	114 (44.2)	1		1	
2-3	375	185 (49.3)	1.2 (0.80-1.9)		0.93 (0.58-1.5)	
4	111	63 (56.8)	1.7 (0.96-2.9)	0.19	1.4 (0.82-2.4)	0.34
**Self-efficacy for contraception[2,***]**						
0-2	117	28 (23.9)	1		1	
3-4	626	334 (53.4)	3.6 (2.4-5.5)	<0.0001	2.4 (1.5-4.1)	0.002
**Timing of most recent sexual activity**						
Within past week	48	24 (50.0)	1			
Within past month	207	110 (53.1)	1.1 (0.58-2.2)			
Within past year	489	228 (46.6)	0.87 (0.47-1.6)	0.42		
**Number of living children[2]**						
No children	638	321 (50.3)	1		1	
1 child or more	106	41 (38.7)	0.62 (0.44-0.89)	0.01	0.57 (0.39-0.85)	0.008

[£] Numbers and percentages may not match exactly because the analysis used sampling weights to account for the sampling design.
OR: odds ratio;
CI: confidence interval; p-value from design based Wald test.

Adjusted ORs: 1 - Adjusted for age and religion. 2 - Adjusted for age, religion, highest education level achieved, currently in education and socioeconomic position.

***** Scored based on the responses to the following five questions: (1) preventing unintended pregnancies is a benefit of contraception, (2) preventing abortions is a benefit of contraception, (3) some contraceptive methods reduce sexually transmitted infections/HIV, (4) modern contraception can help with child spacing, and (5) using modern contraception can allow a woman to complete her education, take up better economic opportunities and fulfil her potential.

****** Scored based on the responses to the following four questions: (1) use of a long-acting reversible contraceptive can make adolescent women permanently infertile, (2) changes to normal menstrual bleeding patterns, which is caused by some contraceptives, are harmful to health, (3) modern contraceptives can make adolescent women permanently fat, and (4) adolescent women who use family planning/birth spacing may become promiscuous.

For peer review only - http://bmjopen.bmj.com/site/about/guidelines.xhtml

For peer review only

******* Scored based on the responses to the following four questions: (1) felt able to start a conversation with her partner about contraception, (2) felt able to use a method of contraception even if her partner did not want her to, (3) felt able to obtain information on contraception services and products if she needed to, and (4) felt able to obtain a contraceptive method if she decided to use one.

For peer review only

For peer review only - http://bmjopen.bmj.com/site/about/guidelines.xhtml

DISCUSSION

In this paper, we describe differences in modern contraceptive use among adolescecnt women aged 15 to 19 years taking part in the A360 evaluation baseline survey according to their marital status. We also present determinants for modern contraception among unmarried sexually active women aged 15 to 19 years who were enrolled in this study.

We found more married women (99.0%) than their unmarried counterparts (79.6%) were out of the formal educational system. In contrast, more unmarried women (52.7%) than married women (27.4%) had achieved secondary school education. These findings highlights an inverse relationship between child marriage, which is prevalent in Tanzania,[17 18] and education achievements. Child marriage is known to impact negatively on educational attainment by adolescent women and perpetuates a vicious cycle of poverty at individual, family and community levels.[1 9] However, child marriage is increasingly acknowledged as a violation of girls' human rights which must be protected by family, community and government authorities.[18]

In this study, although both married and unmarried sexually active women showed similar levels of knowledge about contraception (56.2% vs 62.4%) and self-efficacy for modern contraception use (53.0% vs 56.7%), the proportion of modern contraceptive users was far lower among married women when compared to unmarried women (19.4% vs 48.7%). Generally, in sub Saharan Africa, modern contraception use has remained low despite the rising awareness and knowledge.[5] However, the observed disparities could be pointing to presence of other factors that may have a substantial inhibitive role to contraceptive uptake particularly among married adolescent women. One of those determinants is the social pressure for adolescent women to prove their fertility immediately after marriage, [10 19] which has also been shown to be used as an anchor to win over husband's respect and stabilise marital relationship.[5 20] In this context, proving own fertility, carries

For peer review only - http://bmjopen.bmj.com/site/about/guidelines.xhtml

more weight and higher priority over higher contraceptive knowledge and self efficacy in deciding whether or not to use modern contraception.

The most commonly used contraceptive methods were implants (38.5%) among married women and male condoms (71.6%) among unmarried sexually active women. In a context of held misconceptions particularly against hormonal-based contraceptives for their perceived interference on fertility, this finding might reflect an attempt by unmarried sexually active women to preserve their fertility by opting for non-hormonal based and/or non-invasive methods. Other studies have reported that some adolescent women have chosen unsafe clandestine abortions over hormonal-based contraception.[5 21]. This finding may also reflect that unmarried sexually active adolescent women could have better access to male condoms compared to other methods. In addition, it could reflect the level of exposure and frequency of sexual intercourse, and the type of sexual relationships for unmarried adolescent women which may not require a long-term contraception method. Despite the limitation of condoms being male controlled, the double role of condoms in preventing unintended pregnancies and sexually transmitted infections including HIV, stresses the need for making condoms more accessible and advocating for their proper and regular use among unmarried sexually active adolescent women[7].

Modern contraception use among unmarried sexually active women in the study population was associated with increasing age, increasing levels of education, being in education, hearing of modern contraception from interpersonal sources and in the media in past 12 months, perceiving partner and/or friend support for contraceptive use, as well as higher knowledge about contraception and self efficacy for contraception.

We found that the odds for modern contraception use were low in the respondents who perceived that their partner and/or friends did not approve of their contraception practice compared to those

For peer review only - http://bmjopen.bmj.com/site/about/guidelines.xhtml

who perceived that their partner and/or friends did approve. Social network support has been consistently shown to influence women's decision to use contraception in various age groups and socio-cultural contexts including in sub-Saharan Africa. [5 22 23] We did not observe an association between perception of mothers support for contraceptive use and use of modern contraceptives suggesting that for unmarried adolescent women, partners and friends may be more important influencers than mothers. In addition, we found that, exposure to information about contraception from interpersonal sources or in the media in past 12 months were associated with increased odds for using modern contraception. These findings call for a need for family planning programmes to target the entire community in order to raise awareness of modern contraception and most importantly to engage male partners in support for the uptake of modern contraception.[23]

Among unmarried sexually active women, higher knowledge and self-efficacy for contraceptive use was associated with increased odds for contraceptive use. This finding, when viewed together with other significant determinants such as advancing age and being in education, underscores the spill-over effect of girls' schooling in delaying early marriage as well as its importance in giving adolescent women more time for mental and physical maturity before embarking on sexual and reproductive roles.[9] Additionally, being in education has a potential role to overcome held misconceptions against modern contraception use.[24] This agrees with another study done in rural Mwanza which found that having low education was a risk factor for unplanned pregnancy in young women. [25]

Among unmarried women, the odds for using modern contraception were found to be significantly lower among those with one or more living children when compared to those without. This being a cross sectional analysis, it could partly be telling that the unmarried women with living children are not using contraceptives in the first place hence risking early pregnancies. But it could also be telling us that unmarried women with living children are more likely to be young, out of school, with little

For peer review only - http://bmjopen.bmj.com/site/about/guidelines.xhtml

exposure to information about contraception and low self efficacy to contraception, hence low contraception use. [25 26] In addition, this finding could be pointing to the negative role of mental health issues including depression facing unmarried and/or out of school teenage mothers.[26] Despite having few studies from low and middle income countries, depression has been shown to be an independent risk factor for repeated teenage pregnancy. [27]

Strengths and limitations

In this study, we focused on adolescent women aged 15 – 19 years, a population that is often excluded or underrepresented in most of the studies on modern contraception. We also used the probability sampling approach to interview 3,511 adolescent women from 34 streets in the 15 urban and semi-urban wards of Ilemela district, Mwanza. Therefore, while it may not be possible to generalize our findings to the wider population of adolescent women aged 15 to 19 years living in urban and semi-urban wards of other regions in Tanzania, the sampling approach used allows us to generalise our findings to the wider population of adolescent women aged 15 to 19 years living in urban and semi-urban wards of Ilemela district.

However, this study has some limitations that need to be noted. The cross-sectional design makes temporal causal relationships hard to establish and reverse causality is likely, for instance, use of contraceptives may lead to higher knowledge about contraceptives.

Another limitation was the response rate of potentially eligible women which was relatively low (68.6%), mainly due to young women being at school, even after three visits in attempt to hold interviews. However, it is worth noting that the low response was not due to methodological flaws and it was more likely to involve unmarried women who were in school.

Also, the sample size of married women was small, limiting statistical power to identify determinants for contraceptive use in this group and hence we decided to drop them from the regression analysis.

Lastly, we did not specifically ask the adolescent women whether they were planning on getting pregnant shortly, hence we have no data on this potential explanatory variable. In addition, pregnant women were not asked about contraception as they were not "at risk of pregnancy", the

For peer review only - http://bmjopen.bmj.com/site/about/guidelines.xhtml

same applied to those in post-partum amenorrhea. Therefore, we don't have outcome data for these two subgroups. These are fertility characteristic variables that could make a difference primarily between married and unmarried adolescent women.

CONCLUSION

In Northwest Tanzania, among married and unmarried sexually active women aged 15 to 19 years, we found strong evidence of differences in use of modern contraceptive methods according to marital status of adolescent women.

Among unmarried sexually active women, contraceptive use was significantly associated with increasing age, increasing levels of education, being in education, hearing information on modern contraception from interpersonal sources and in the media in the past 12 months, perceiving partner and/or friend support for contraception use, as well as higher knowledge and self efficacy for modern contraception.

In order to optimise their impact, sexual and reproductive health programmes aiming to increase uptake of modern contraceptives should consider the importance of being in education and social support for contraceptive use among adolescent women. Hence the need to focus intervention efforts on more vulnerable unmarried sexually active adolescent women particularly those with lower education/socioeconomic status and/or those who are already teenage mothers.

CONFLICTING INTERESTS:

The authors declare that they have no competing interests.

FUNDING:

The Bill & Melinda Gates Foundation and the Children’s Investment Fund Foundation. The funding bodies had no role in the design of the study and collection, analysis, and interpretation of data and in writing the manuscript.

For peer review only - http://bmjopen.bmj.com/site/about/guidelines.xhtml

AUTHORS' CONTRIBUTIONS:

MKN, CJA, SK, CB and AMD were involved in conception and study design. CB provided statistical expertise. MKN and CJA were involved in drafting of the manuscript. SK, CB and AMD were involved in critical revision of the manuscript for important intellectual content. All the authors were involved in final approval of the manuscript and decision to submit the manuscript for publication.

ACKNOWLEDGEMENTS:
We would like to thank all study participants for their participation and the A360 baseline study team for their dedicated work during data collection.

We also thank Itad as the lead organisation responsible for the overall A360 evaluation. Avenir Health as a partner in the overall A360 evaluation. PSI Headquarters and PSI Tanzania for their support with site selection and engagement in conversation regarding the design.

DATA AVAILABILITY

Individual de-identified data used for this analysis are available from AMD Aoife.Doyle@lshtm.ac.uk on reasonable request.

TABLE LEGEND

Table 1: Characteristics of married and unmarried-sexually active women aged 15-19 years in Mwanza, Tanzania[1].

Table 2: Prevalence of contraceptive use among women aged 15–19 years in Mwanza, Tanzania by marital status[1].

Table 3: Factors associated with modern contraception use among unmarried sexually active women aged 15–19 years in Mwanza, Tanzania, (N=744)[£]

For peer review only - http://bmjopen.bmj.com/site/about/guidelines.xhtml

REFERENCES

1. The World Health Organisation guidelines on preventing early pregnancy and poor reproductive outcomes among adolescents in developing countries. Geneva: WHO, 2011.

2. Blum RW, Gates WH. Girlhood, not motherhood: preventing adolescent pregnnancy. New York: United Nations Populations Fund (UNFPA), 2015.

3. Family planning 2020 initiative [cited 2018 October]. Available from: https://www.familyplanning2020.org/measurement-hub#global-data accessed September 2018.

4. The UN 2030 agenda for sustainable development New York: United Nations; 2015 [cited 2019 Jan]. Available from: https://sustainabledevelopment.un.org/content/documents/21252030%20Agenda%20for%20Sustainable%20Development%20web.pdf accessed October 2018.

5. Blackstone SR, Nwaozuru U, Iwelunmor J. Factors Influencing Contraceptive Use in Sub-Saharan Africa: A Systematic Review. *Int Q Community Health Educ* 2017;37(2):79-91. doi: 10.1177/0272684X16685254

6. Nalwadda G, Mirembe F, Byamugisha J, et al. Persistent high fertility in Uganda: young people recount obstacles and enabling factors to use of contraceptives. *BMC Public Health* 2010;10:530. doi: 10.1186/1471-2458-10-530

7. Williamson LM, Parkes A, Wight D, et al. Limits to modern contraceptive use among young women in developing countries: a systematic review of qualitative research. *Reprod Health* 2009;6(3):3. doi: 10.1186/1742-4755-6-3

8. Conde-Agudelo A, Belizan JM, Lammers C. Maternal-perinatal morbidity and mortality associated with adolescent pregnancy in Latin America: Cross-sectional study. *Am J Obstet Gynecol* 2005;192(2):342-9. doi: 10.1016/j.ajog.2004.10.593

For peer review only - http://bmjopen.bmj.com/site/about/guidelines.xhtml

9. Wodon Q, Nayihouba A, Onagoruwa A, et al. Economic impacts of child marriage: global synthesis report. Washington DC: International Centre for Research on Women (ICRW), 2017:43-49.

10. Coll CVN, Ewerling F, Hellwig F, et al. Contraception in adolescence: the influence of parity and marital status on contraceptive use in 73 low-and middleincome countries *Reproductive Health* 2019(16):21-33. doi: 10.1186/s12978-019-0686-9 [published Online First: 21 Feb 2019]

11. Atchison CJ, Mulhern E, Kapiga S, et al. Evaluating the impact of an intervention to increase uptake of modern contraceptives among adolescent girls (15 - 19 years) in Nigeria, Ethiopia and Tanzania: the adolescents 360 quasi-experimental study protocol. *BMJ Open* 2018;8(5):e021834. doi: 021810.021136/bmjopen-022018-021834

12. Kavishe B, Biraro S, Baisley K, et al. High prevalence of hypertension and of risk factors for non-communicable diseases (NCDs): a population based cross-sectional survey of NCDS and HIV infection in Northwestern Tanzania and Southern Uganda. *BMC Med* 2015;13(1):126. doi: 10.1186/s12916-015-0357-9

13. Ministry of Health (MoHCDGEC) [Tanzania Mainland], Ministry of Health (MoH) [Zanzibar], National Bureau of Statistics (NBS), et al. Tanzania Demographic and Health Survey and Malaria Indicator Survey (TDHS-MIS) 2015-16. Dar es Salaam, Tanzania.: National Bureau of Statistics (NBS), 2016.

14. Central Statistical Agency (CSA) [Ethiopia], and ICF. Ethiopia Demographic and Health Survey 2016. Addis Ababa, Ethiopia and Rockville, Maryland, USA: Central Statistical Agency [Ethiopia], 2016.

15. World Health Organisation Family Planning Fact Sheet [Available from: https://www.who.int/news-room/fact-sheets/detail/family-planning-contraception accessed 09 March 2019.

16. Tanzania Equity Tool [cited 2019 16 Jan]. Available from: http://www.equitytool.org/tanzania/ accessed 18 Jan 2019.

For peer review only - http://bmjopen.bmj.com/site/about/guidelines.xhtml

17. Koski A, Clark S, Nandi A. Has child marriage declined in sub-Saharan Africa? An analysis of trends in 31 countries. *Popul Dev Rev* 2017;43(1):7 - 29.

18. (Tanzania) MoH. National survey on the drivers and consequences of child marrige in Tanzania. Dar es Salaam - Tanzania: Ministry of Health (MoHCDGEC), 2017.

19. UNDESA. Trends in the contraceptive use worldwide. New York: United Nations Department of Economic and Social Affairs (UNDESA - Population Division). 2015.

20. Eaton L, Flisher AJ, Aar LE. Unsafe sexual behaviour in south African youth. *Soc Sci Med* 2002;56(2003):149 - 65.

21. Plummer ML, Wamoyi J, Nyalali K, et al. Aborting and suspending pregnancy in rural Tanzania: an ethnography of young people's beliefs and practices. *Stud Fam Plann* 2008;39(4):281-92.

22. Omideyi AK, Akinyemi AI, Aina OI, et al. Contraceptive practice, unwanted pregnancies and induced abortion in southwest Nigeria. *Global public health* 2011;6 Suppl 1:S52-72. doi: 10.1080/17441692.2011.594073

23. Samandari G, Speizer IS, O'Connell K. The role of social support and parity on contraceptive use in Cambodia. *Int Perspect Sex Reprod Health* 2010;36(3):122-31.

24. Mardi A, Ebadi A, Shahbazi S, et al. Factors influencing the use of contraceptives through the lens of teenage women: a qualitative study in Iran. *BMC Public Health* 2018;18(1):202. doi: 10.1186/s12889-018-5116-3

25. Calvert C, Baisley K, Doyle AM, et al. Risk factors for unplanned pregnancy among young women in Tanzania. *J Fam Plann Reprod Health Care* 2013;39(4):e2. doi: 10.1136/jfprhc-2012-100389

26. SmithBattle L, Freed P. Teen Mothers' Mental Health. *MCN The American journal of maternal child nursing* 2016;41(1):31-6; quiz E3-4. doi: 10.1097/NMC.0000000000000198

27. Maravilla JC, Betts KS, e Cruz C, et al. Factors influencing repeated teenage pregnancy: A review and meta analysis. *Am J Obstet Gynecol* 2017;19e

For peer review only

For peer review only - http://bmjopen.bmj.com/site/about/guidelines.xhtml

Reporting checklist for cross sectional study.

Based on the STROBE cross sectional guidelines.

Instructions to authors

Complete this checklist by entering the page numbers from your manuscript where readers will find each of the items listed below.

Your article may not currently address all the items on the checklist. Please modify your text to include the missing information. If you are certain that an item does not apply, please write "n/a" and provide a short explanation.

Upload your completed checklist as an extra file when you submit to a journal.

In your methods section, say that you used the STROBE cross sectional reporting guidelines, and cite them as:

von Elm E, Altman DG, Egger M, Pocock SJ, Gotzsche PC, Vandenbroucke JP. The Strengthening the Reporting of Observational Studies in Epidemiology (STROBE) Statement: guidelines for reporting observational studies.

		Reporting Item	Page Number
Title	#1a	Indicate the study's design with a commonly used term in the title or the abstract	1 & 2
Abstract	#1b	Provide in the abstract an informative and balanced summary of what was done and what was found	2

For peer review only

Background / rationale	#2	Explain the scientific background and rationale for the investigation being reported	4 & 5
Objectives	#3	State specific objectives, including any prespecified hypotheses	5
Study design	#4	Present key elements of study design early in the paper	5
Setting	#5	Describe the setting, locations, and relevant dates, including periods of recruitment, exposure, follow-up, and data collection	5
Eligibility criteria	#6a	Give the eligibility criteria, and the sources and methods of selection of participants.	6
	#7	Clearly define all outcomes, exposures, predictors, potential confounders, and effect modifiers. Give diagnostic criteria, if applicable	8 - 10
Data sources / measurement	#8	For each variable of interest give sources of data and details of methods of assessment (measurement). Describe comparability of assessment methods if there is more than one group. Give information separately for exposed and unexposed groups if applicable.	8 - 9
Bias	#9	Describe any efforts to address potential sources of bias	6 & 7
Study size	#10	Explain how the study size was arrived at	7
Quantitative variables	#11	Explain how quantitative variables were handled in the analyses. If applicable, describe which groupings were chosen, and why	8 - 10

For peer review only

Statistical methods	#12a	Describe all statistical methods, including those used to control for confounding	9
	#12b	Describe any methods used to examine subgroups and interactions	
	#12c	Explain how missing data were addressed	
	#12d	If applicable, describe analytical methods taking account of sampling strategy	9
	#12e	Describe any sensitivity analyses	
Participants	#13a	Report numbers of individuals at each stage of study—eg numbers potentially eligible, examined for eligibility, confirmed eligible, included in the study, completing follow-up, and analysed. Give information separately for for exposed and unexposed groups if applicable.	11
	#13b	Give reasons for non-participation at each stage	11
	#13c	Consider use of a flow diagram	n/a
Descriptive data	#14a	Give characteristics of study participants (eg demographic, clinical, social) and information on exposures and potential confounders. Give information separately for exposed and unexposed groups if applicable.	11
	#14b	Indicate number of participants with missing data for each variable of interest	11,12,13
Outcome data	#15	Report numbers of outcome events or summary measures.	14

For peer review only

		Give information separately for exposed and unexposed groups if applicable.	
Main results	#16a	Give unadjusted estimates and, if applicable, confounder-adjusted estimates and their precision (eg, 95% confidence interval). Make clear which confounders were adjusted for and why they were included	17- 19
	#16b	Report category boundaries when continuous variables were categorized	11 , 12
	#16c	If relevant, consider translating estimates of relative risk into absolute risk for a meaningful time period	n/a
Other analyses	#17	Report other analyses done—e.g., analyses of subgroups and interactions, and sensitivity analyses	n/a
Key results	#18	Summarise key results with reference to study objectives	23
Limitations	#19	Discuss limitations of the study, taking into account sources of potential bias or imprecision. Discuss both direction and magnitude of any potential bias.	3 & 24
Interpretation	#20	Give a cautious overall interpretation considering objectives, limitations, multiplicity of analyses, results from similar studies, and other relevant evidence.	25
Generalisability	#21	Discuss the generalisability (external validity) of the study results	3 & 24
Funding	#22	Give the source of funding and the role of the funders for the present study and, if applicable, for the original study on which	25

For peer review only

the present article is based

The STROBE checklist is distributed under the terms of the Creative Commons Attribution License CC-BY. This checklist can be completed online using https://www.goodreports.org/, a tool made by the EQUATOR Network in collaboration with Penelope.ai

For peer review only
